# HDAC2 promotes autophagy-associated HCC malignant progression by transcriptionally activating LAPTM4B

**DOI:** 10.1038/s41419-024-06981-3

**Published:** 2024-08-15

**Authors:** Meifeng Wang, Jianping Liao, Jie Wang, Meifang Xu, Ye Cheng, Lixin Wei, Aimin Huang

**Affiliations:** 1 Department of Pathology, School of Basic Medical Sciences, Fujian Medical University, 88 Jiaotong Road, Fuzhou, Fujian 350004 China; 2 Institute of Oncology, Fujian Medical University, 88 Jiaotong Road, Fuzhou, Fujian 350004 China; 3 Tumor Immunology and Gene Therapy Center, Third Affiliated Hospital of Second Military Medical University, 225 Changhai Road, Shanghai, 200438 China

**Keywords:** Cancer, Oncogenes

## Abstract

Hepatocellular carcinoma (HCC) is a significant global health challenge. The activation of autophagy plays an essential role in promoting the proliferation and survival of cancer cells. However, the upstream regulatory network and mechanisms governing autophagy in HCC remain unclear. This study demonstrated that histone deacetylase 2 (HDAC2) regulates autophagy in HCC. Its expression was elevated in HCC tissues, and high HDAC2 expression was strongly associated with poor prognosis in individuals with HCC. Integrated in vitro and in vivo investigations confirmed that HDAC2 promotes autophagy and autophagy-related malignant progression in HCC. Mechanistically, HDAC2 bound specifically to the lysosome-associated protein transmembrane 4-β (LAPTM4B) promoter at four distinct binding sites, enhancing its transcriptional activation and driving autophagy-related malignant progression in HCC. These findings establish LAPTM4B as a direct target gene of HDAC2. Furthermore, the selective inhibitor of HDAC2 effectively alleviated the malignant development of HCC. In addition, multivariate Cox regression analysis of 105 human HCC samples revealed that HDAC2 expression is an independent predictor of HCC prognosis. This study underscores the crucial role of the HDAC2-LAPTM4B axis in regulating autophagy in the malignant evolution of HCC and highlights the potential of targeting HDAC2 to prevent and halt the malignant progression of HCC.

## Introduction

Autophagy is a classic lysosome-dependent process that degrades damaged cytoplasmic proteins, macromolecules and organelles [1]. Studies have confirmed that during the initial stages of tumorigenesis, autophagy can exert an antitumor effect by promoting genomic stability and maintaining the integrity of the intracellular environment [2]. However, as tumor progresses, autophagy facilitates malignant progression by providing energy and nutrients, stimulating angiogenesis, and promoting tumor growth [3, 4]. Hepatocellular carcinoma (HCC) is the third leading cause of cancer-related deaths worldwide and one of the most lethal cancers [5]. Substantial evidence suggests that autophagy drives the malignant progression of solid tumors, including HCC [6–8]. Most HCC patients are diagnosed in advanced stages, where tumors have greater energy demands, rendering autophagy more tumor-promoting. Therefore, inhibiting autophagy is a promising strategy for antitumor treatment. A comprehensive understanding of the upstream regulators of autophagy and its regulatory mechanisms in HCC will enhance our knowledge of the intrinsic mechanisms underlying HCC development and offer new insights and potential therapeutic targets for HCC.

Histone deacetylase 2 (HDAC2), a member of the class I HDACs family, is an enzyme with high activity and selectivity towards histone substrates. HDAC2 is broadly expressed in various human tissues, with the lowest expression found in the liver. Studies have shown that HDAC2 expression is significantly higher in HCC tissues compared to normal liver tissues, and elevated HDAC2 expression is strongly associated with poor prognosis in HCC patients [9]. Initial experiments revealed that HDAC2 is responsible for the deacetylation of histone H3 and H4 N-termini, thereby inhibiting the transcription of downstream genes [10]. It has been reported that HDAC2 prevents apoptosis and accelerates cell cycle progression by inhibiting p53 and promoting MYC expression, contributing to malignancy [11, 12]. Moreover, increasing evidence suggests that HDAC2, as an oncogene, induces cell cycle alterations and promotes HCC cell proliferation, playing a crucial role in HCC malignant progression [13]. Numerous studies have also demonstrated that HDAC2 inhibitors exhibit pronounced antitumor effects [14–16]. However, whether HDAC2 promotes autophagy and the associated malignant progression in HCC, as well as the underlying regulatory mechanisms, remain unclear.

Lysosome-associated protein transmembrane 4-β (LAPTM4B) belongs to the LAPTM family and contains four transmembrane structural regions and a typical lysosomal localization signal [17]. It is a well-recognized oncogene, strongly expressed in various cancers, including ovarian, bladder, breast, and lung cancers [18–21]. Previous research has found that LAPTM4B-35 is extensively expressed in HCC tissues and is associated with TNM stage, invasion and metastasis, and postoperative survival of patients [22, 23]. LAPTM4B has been established as crucial for the regulation of autophagy. In human HCC, LAPTM4B amplifies autophagic signals through ATG3 [24]. In addition, it has been demonstrated that the absence of LAPTM4B in HCC cells prevents autophagosome-lysosome fusion and autolysosome formation under starvation conditions [25]. However, the upstream regulatory mechanism of LAPTM4B in promoting autophagy in HCC has not been elucidated.

In this study, we demonstrated that HDAC2, which is significantly expressed in tissues from HCC patients, serves as an independent predictor of patient outcomes. HDAC2 promoted autophagy and autophagy-related malignant progression of HCC by transcriptionally activating LAPTM4B. We found that HDAC2 directly binds to four specific binding sites on the LAPTM4B promoter to perform these functions. These findings identify LAPTM4B as a novel direct target gene of HDAC2. Importantly, we also discovered that selective HDAC2 inhibitor effectively ameliorates HCC malignancy by reducing tumor volume, significantly inhibiting HCC tumor formation and attenuating HCC autophagy in vivo. Our research reveals the critical role of the HDAC2-LAPTM4B axis in HCC malignant progression through the regulation of autophagy, suggesting that HDAC2 could be a promising target for HCC treatment.

## Results

### HDAC2 is upregulated in HCC tissues

**Fig. 1:**
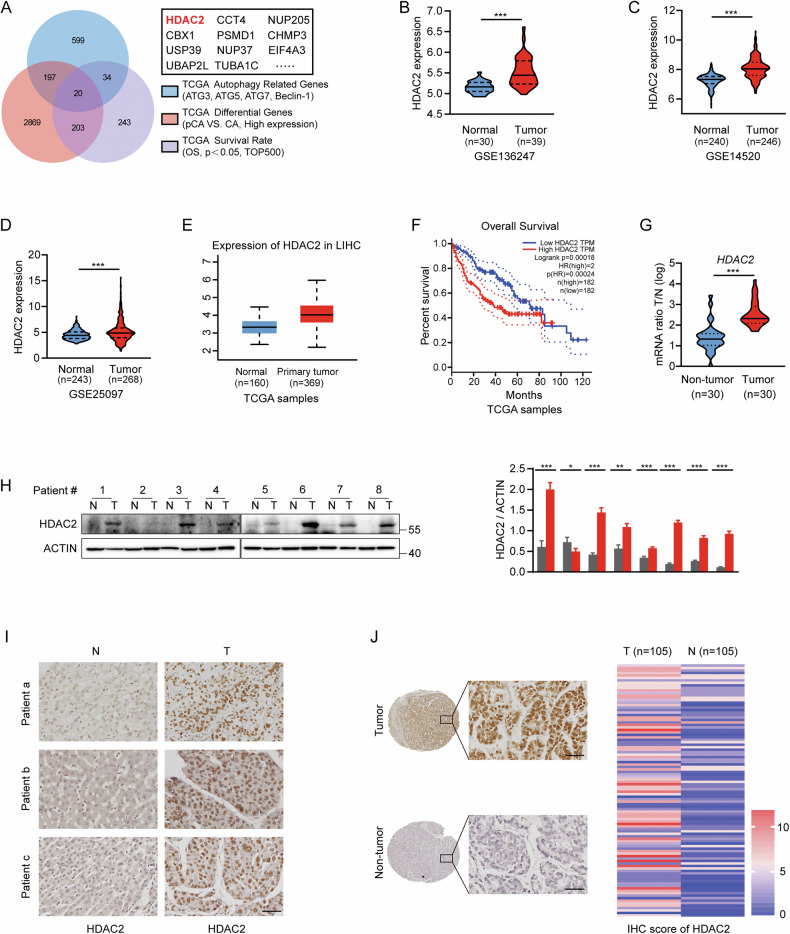
Upregulation of HDAC2 is frequently noted in HCC tissues. **A** Integrative analysis of the genes associated with autophagy key marker (ATG3, ATG5, ATG7, and Beclin-1), the highly expressed genes and genes related to adverse outcome in HCC from The Cancer Genome Atlas (TCGA) database. **B**–**E** Expression of HDAC2 mRNA in the Gene Expression Omnibus (GEO) datasets (GSE136247, GSE14520, GSE25097) and TCGA database. **F** Kaplan–Meier survival analysis (KMSA) of overall survival (OS) of HCC patients based on HDAC2 expression in the TCGA database (log-rank test). **G** qRT-PCR examination of HDAC2 mRNA expression in thirty HCC and nearby nontumor tissues. **H** Western blotting assay of HDAC2 protein expression in eight HCC and nearby nontumor tissues. **I** Immunohistochemistry (IHC) staining of HDAC2 in three representative HCC tissues and adjacent normal tissues. Scale bar, 50 µm. **J** Representative IHC images of HDAC2 using HCC tissue microarrays (TMAs) and the IHC score of HDAC2. Scale bar, 50 µm. All data were subjected to at least three separate experiments. Data were displayed as mean ± SD. Unpaired Student’s t tests were employed for two-variable comparisons. *p < 0.05; **p < 0.01; ***p < 0.001.

To deeply investigate the regulatory mechanisms of autophagy in HCC, we conducted a comprehensive analysis of genes associated with key autophagy markers (ATG3, ATG5, ATG7, and Beclin-1), genes highly expressed in HCC, and genes linked to poor outcomes in HCC using data from The Cancer Genome Atlas (TCGA) database. This analysis revealed 20 genes, including HDAC2, that overlapped (Fig. 1A). Further evaluation of Gene Expression Omnibus (GEO) datasets (GSE136247, GSE14520, GSE25097) and the TCGA database highlighted significantly elevated levels of HDAC2 in HCC tissues, with higher HDAC2 expression correlating with poorer prognoses (Fig. 1B–F). To corroborate these findings, we assessed HDAC2 mRNA levels in thirty HCC tissues and paired normal tissues using quantitative real-time PCR (qRT-PCR) (Fig. 1G). In addition, HDAC2 protein expression in HCC tissues was examined using western blotting in eight cases and immunohistochemistry (IHC) staining in three cases (Fig. 1H, I). These results were consistent with the date from the databases. Finally, IHC staining of tissue microarrays (TMAs) containing 105 HCC specimens further verified the upregulation of HDAC2 protein (Fig. 1J). Collectively, these date indicate that HDAC2 expression is remarkably elevated in HCC tissues.

### HDAC2 drives autophagy in HCC tumor cells

**Fig. 2:**
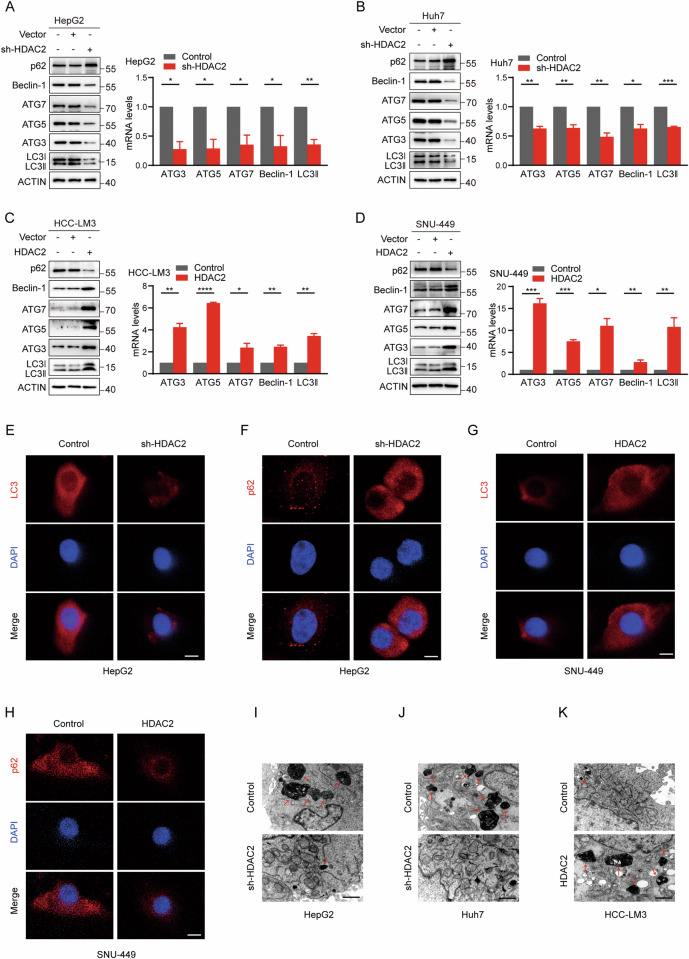
HDAC2 promotes autophagy in HCC tumor cells. **A**, **B** Western blotting and qRT-PCR assays of ATG3, ATG5, ATG7, Beclin-1, LC3II, and p62 levels in HepG2 and Huh7 cells with or without HDAC2-knockdown. **C**, **D** Western blotting and qRT-PCR assays of ATG3, ATG5, ATG7, Beclin-1, LC3II, and p62 levels in control cells and HDAC2 overexpressed HCC-LM3 and SNU-449 cells. **E**, **F** Immunofluorescence (IF) staining of LC3 and p62 in HepG2 cells with HDAC2 knockdown or not. Red puncta signify LC3 or p62 and blue puncta signify DAPI. Scale bar, 50 µm. **G**, **H** IF staining of LC3 and p62 in control cells and HDAC2 overexpressed SNU-449 cells. Red puncta signify LC3 or p62 and blue puncta signify DAPI. Scale bar, 50 µm. **I**, **J** Transmission electron microscopy (TEM) assay showing autophagosomes in HepG2 and Huh7 cells with or without HDAC2-knockdown. Scale bar, 1 µm. **K** TEM demonstrating autophagosomes in HCC-LM3 cells with HDAC2 overexpression or not. Scale bar, 1 µm. All data were subjected to at least three separate experiments. Data were displayed as mean ± SD. Unpaired Student’s t tests were employed for two-variable comparisons. *p < 0.05; **p < 0.01; ***p < 0.001; ****p < 0.0001.

To clarify the function of HDAC2 in autophagy, we conducted a series of cytological investigations. We began by examining HDAC2 expression in seven human HCC cell lines (Fig. S1A–C) and then established HDAC2-silenced HepG2 and Huh7 cells, along with HDAC2-overexpressing HCC-LM3 and SNU-449 cells, demonstrating knockdown efficiencies greater than 70% and overexpression efficiencies exceeding three-fold (Fig. S1D–G). Inhibition of HDAC2 expression resulted in a reduction of autophagy-related genes ATG3, ATG5, ATG7, Beclin-1, and LC3II, along with increased expression of p62, whereas HDAC2 overexpression had the opposite effect, as confirmed by qRT-PCR and western blotting (Fig. 2A–D). Consistently, Immunofluorescence (IF) assays showed that HDAC2 knockdown led to a significant reduction in LC3 dot staining and increased p62 expression, while HDAC2 overexpression produced inverse results (Fig. 2E–H and Fig. S1H–K). Transmission electron microscopy (TEM) examination revealed that the formation of double-membrane capsules, characteristic of autophagy, was reduced in HDAC2-deficient HCC tumor cells (Fig. 2I, J), and the number of autophagosomes significantly increased following HDAC2 overexpression (Fig. 2K). These results support the notion that HDAC2 is essential for maintaining autophagy in HCC tumor cells.

### HDAC2 promotes autophagy-associated HCC malignancy

**Fig. 3:**
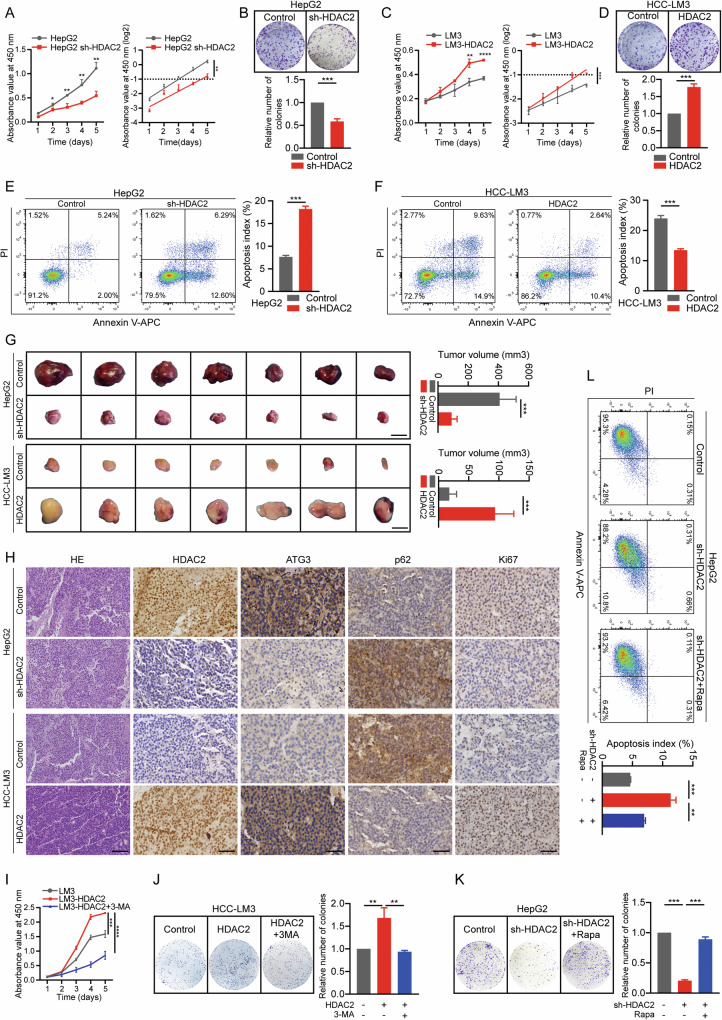
HDAC2 promotes autophagy-associated HCC malignancy. **A**, **C** (Left) Cell Counting Kit-8 (CCK-8) assays in HepG2 and HCC-LM3 cells with HDAC2 knockdown or overexpression, respectively. (Right) Cell proliferation curves based on OD values at 450 nm transformed to log2 scale. **B**, **D** (Top) Representative colony formation photos of HCC cells with HDAC2 knockdown or overexpression. (Bottom) Quantification of colonies formed. **E**, **F** Cell apoptosis assays in HepG2 and HCC-LM3 cells with HDAC2 knockdown or overexpression, respectively. **G** Subcutaneous xenograft experiment evaluating tumor formation capacity of HepG2 and HCC-LM3 cells with HDAC2 knockdown or overexpression, respectively (*n* = 7 per group). Scale bar, 5 mm. Tumor volumes were measured. **H** Representative graphs of IHC staining showing expression levels of HDAC2, ATG3, p62, and Ki67 in xenografted tumors formed from HepG2 and HCC-LM3 cells knocking down or overexpressing HDAC2. Scale bar, HE: 100 µm, others: 50 µm. **I** CCK-8 assays in HCC-LM3 cells with or without HDAC2 overexpression. The autophagy inhibitor 3-MA was employed to perform salvage assays. **J**, **K** (Left) Representative colony formation images of HDAC2-overexpression and HDAC2 knockdown HCC cells. 3-MA and Rapa was employed as an autophagy inhibitor and agonists, respectively. (Right) Quantification of colonies formed. **L** Cell apoptosis assays in HepG2 cells with HDAC2 knockdown. Rapa was used as an autophagy agonist. All data were subjected to at least three separate experiments. Data were displayed as mean ± SD. Unpaired Student’s t tests were employed for two-variable comparisons. *p < 0.05; **p < 0.01; ***p < 0.001; ****p < 0.0001.

Given that HDAC2 is crucial for promoting autophagy in HCC and that autophagy is linked to cancer malignancy [26–29], we further examined the tumor-promotive function of HDAC2 and its connection to autophagy. Knockdown of HDAC2 resulted in decreased cell proliferation and clone formation ability compared to controls, as confirmed by colony formation and cell counting kit-8 (CCK-8) assays. In HepG2 and Huh7 cells with HDAC2 knockdown, the doubling time increased by 38.4 h and 7.2 h, and the number of colony clusters was reduced by 40 and 60%, respectively (Fig. 3A, B and Fig. S2A, B). Conversely, cells overexpressing HDAC2 displayed superior proliferation and clone formation ability compared to controls. The doubling time of HCC-LM3 and SNU-449 cells with HDAC2 overexpression was shortened by 45.6 h and 31.2 h, and the number of colony clusters was 1.8 and 1.9 times higher than controls (Fig. 3C, D and Fig. S2C, D). In addition, flow cytometry showed that cells with HDAC2 knockdown exhibited higher apoptotic rates, whereas cells with HDAC2 overexpression showed contrasting results (Fig. 3E, F and Fig. S2E, F). Furthermore, we explored whether HDAC2 promotes carcinogenesis in vivo by injecting HCC cells subcutaneously into nude mice. The development of subcutaneous tumors transfected with LV-sh-HDAC2 was impeded compared to controls (HepG2 cells), whereas transfection with LV-HDAC2 promoted subcutaneous tumorigenesis compared with controls (HCC-LM3 cells) (Fig. 3G). IHC staining of tumor tissue sections showed that subcutaneous tumors transfected with LV-sh-HDAC2 had decreased ATG3 expression, increased p62 expression, and significantly reduced proliferation (Fig. 3H). Conversely, the opposite effect was observed in subcutaneous tumors transfected with LV-HDAC2 (Fig. 3H).

To determine whether HDAC2 promotes HCC malignancy by facilitating autophagy, we administered the autophagy inhibitor 3-methyladenine (3-MA) to HDAC2-overexpressing cells. CCK8, colony formation, and flow cytometry assays showed that 3-MA administration reversed the increase in proliferation and clone formation, as well as the decrease in apoptotic rates in HDAC2-overexpressing cells (Fig. 3I, J and Fig. S2G–J). In HCC-LM3 and SNU-449 cells, HDAC2 overexpression shortened the cell doubling time by 9.6 h and 12 h, respectively, whereas 3-MA treatment prolonged it by 33.6 h and 28.8 h (Fig. 3I and Fig. S2G, H). In addition, the number of clonal clusters induced by HDAC2 was reduced by 44 and 56% with 3-MA treatment (Fig. 3J and Fig. S2I). Consistently, HDAC2-silenced cells treated with the autophagy agonist rapamycin (Rapa) showed remarkable results, as Rapa treatment rescued the attenuation of cell proliferation and clone formation and decreased apoptosis (Fig. 3K, L and Fig. S2K–M). HepG2 and Huh7 cells with HDAC2 knockdown showed an 80 and 41% decrease in the number of clonal clusters, respectively, while Rapa administration increased it by 70 and 46% (Fig. 3K). Altogether, these results indicate that HDAC2 promotes the malignant progression of HCC through autophagy activation.

### HDAC2 upregulates LAPTM4B expression in HCC

**Fig. 4:**
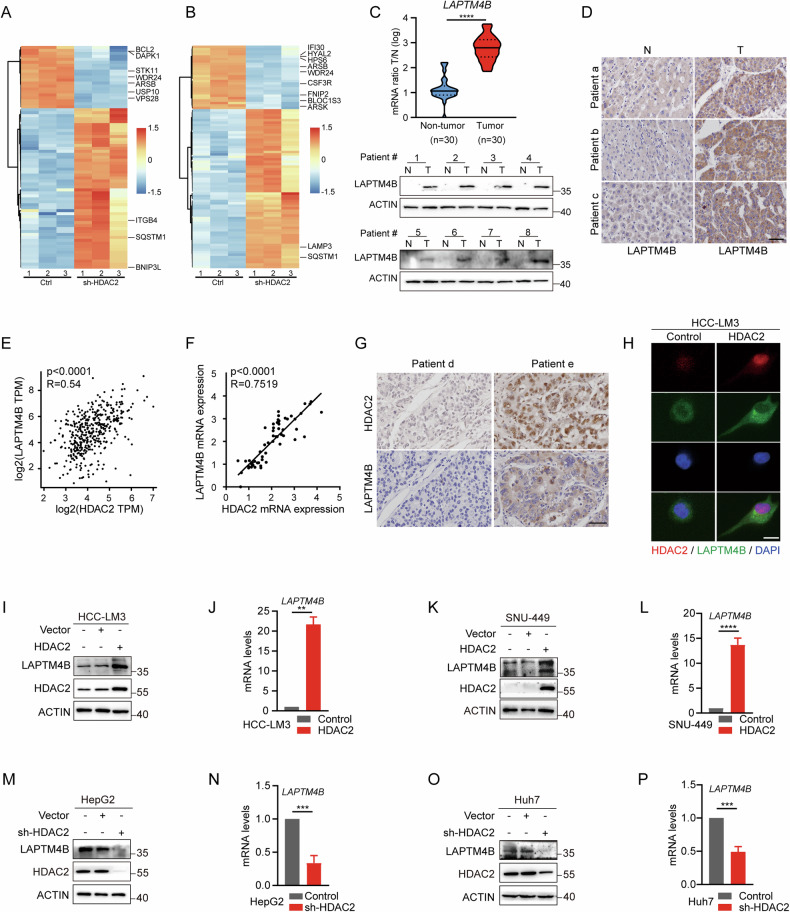
HDAC2 upregulates LAPTM4B expression in HCC. **A** RNA-seq transcriptional profile heatmap of HepG2 cells with and without HDAC2 knockdown, highlighting differentially expressed autophagy-related genes. **B** Heatmap depicting differential expression of lysosome-associated genes after HDAC2 knockdown. **C** (Top) qRT-PCR analysis of LAPTM4B mRNA expression in thirty paired HCC and adjacent nontumor tissues. (Bottom) Western blotting showing LAPTM4B protein expression in eight paired HCC and adjacent nontumor tissues. **D** IHC staining of LAPTM4B in representative HCC tissues and adjacent normal tissues. Scale bar, 50 µm. **E** Pearson’s correlation analysis demonstrating the association between HDAC2 and LAPTM4B expression in HCC patients from the TCGA database. **F** Correlation between mRNA expression levels of HDAC2 and LAPTM4B in thirty paired HCC tissues and adjacent liver tissues. **G** Representative IHC images showing co-expression of HDAC2 and LAPTM4B in HCC tissues. Scale bars, 50 µm. **H** IF staining depicting co-expression pattern of HDAC2 and LAPTM4B in HCC tissues. Scale bar, 50 µm. **I**–**P** qRT-PCR and western blotting analyses of LAPTM4B expression in HDAC2 overexpressing or knockdown HCC cells. All data were subjected to at least three separate experiments. Data were displayed as mean ± SD. Unpaired Student’s t tests were employed for two-variable comparisons. *p < 0.05; **p < 0.01; ***p < 0.001; ****p < 0.0001.

To elucidate the molecular mechanism by which HDAC2 regulates autophagy in HCC, we performed RNA-seq analysis to identify potential HDAC2 target genes. We identified 3396 differentially expressed genes in HDAC2 knockdown HepG2 cells compared to controls, including several autophagy-related genes such as SQSTM1, USP10 [30], VPS28 [31], BCL2 [32, 33], WDR24, DAPK1 [34, 35], STK11 [36], ARSB, and others (Fig. 4A). These findings underscore the significant role of HDAC2 in controlling autophagy in HCC tumor cells. In addition, we identified 91 differentially expressed lysosome-associated genes (IFI30 [37], HYAL2 [38], HPS6, ARSB, WDR24 [39], CSF3R [40], FNIP2 [41], ARSK, and others), indicating that HDAC2 can markedly influence lysosomal pathways (Fig. 4B). Since lysosomes are pivotal in autophagy function [42], we focused on downstream targets of HDAC2 among lysosomal genes. Previous studies have implicated LAPTM4B in regulating autophagy in cancer contexts [25, 43–45]. Based on the RNA-seq results and the indications of HDAC2 and LAPTM4B involvement in promoting autophagy, we hypothesized that HDAC2 may mediate LAPTM4B to promote autophagy in HCC.

HCC tissue specimens and corresponding adjacent normal liver tissues were randomly collected for qRT-PCR, western blotting, and IHC analysis. The results confirmed that LAPTM4B expression was significantly elevated in HCC tissues (Fig. 4C, D). Subsequent analysis of TCGA database revealed a positive correlation between HDAC2 and LAPTM4B expression in HCC (Fig. 4E). Single-cell sequencing data from HCC in the GEO database (GSE125449) further indicated similar expression profiles of HDAC2 and LAPTM4B in HCC cells (Fig. S3A–C). Moreover, mRNA and protein expression levels of HDAC2 and LAPTM4B were highly correlated in seven HCC cell lines and HCC tissues (Fig. 4F–H and Fig. S3D–H), as evidenced by qRT-PCR, western blotting, IHC, and IF staining. Subsequent investigation into the effect of HDAC2 on LAPTM4B expression revealed that LAPTM4B levels were elevated in HDAC2-overexpressing cells and reduced in HDAC2-knockdown cells (Fig. 4I–P). However, the expression of HDAC2 was unremarkably altered by either overexpression or knockdown of LAPTM4B (Fig. S3I–M). Taken together, these findings support the hypothesis that HDAC2 upregulates LAPTM4B expression in HCC.

### HDAC2 regulates LAPTM4B to promote both autophagy and its associated malignancy in HCC

**Fig. 5:**
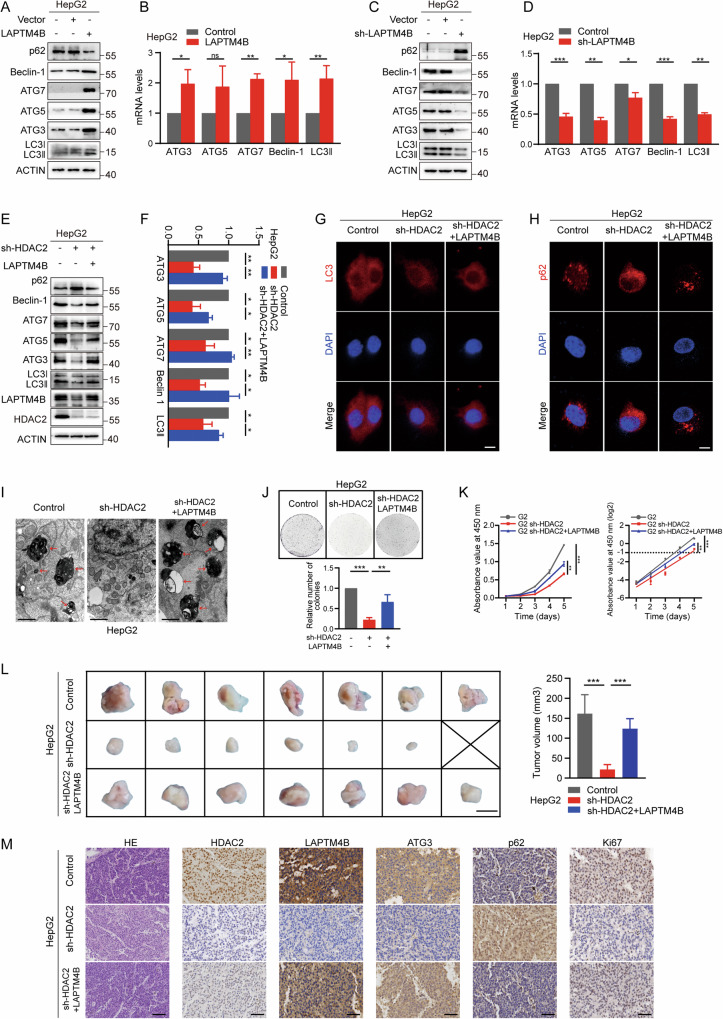
HDAC2 regulates LAPTM4B to promote both autophagy and its associated malignancy in HCC. **A**, **B** qRT-PCR and western blotting assays showing levels of ATG3, ATG5, ATG7, Beclin-1, LC3II, and p62 in HepG2 cells with or without LAPTM4B overexpression. **C**, **D** qRT-PCR and western blotting assays demonstrating levels of ATG3, ATG5, ATG7, Beclin-1, LC3II, and p62 after knockdown of LAPTM4B in HepG2 cells. **E**, **F** Western blotting and qRT-PCR assays measuring levels of ATG3, ATG5, ATG7, Beclin-1, LC3II, p62, LAPTM4B, and HDAC2 in HDAC2-knockdown HepG2 cells with or without LAPTM4B overexpression. **G**, **H** IF staining of LC3 and p62 in HDAC2-knockdown HepG2 cells with or without LAPTM4B overexpression. Red puncta indicate LC3 or p62, and blue puncta indicate DAPI. Scale bar, 50 µm. **I** TEM images showing autophagosomes in HDAC2-knockdown HepG2 cells with or without LAPTM4B overexpression. Scale bar, 1 µm. **J** (Top) Representative images of colony formation assays in HDAC2-knockdown HepG2 cells with or without LAPTM4B overexpression. (Bottom) Quantification of colonies formed. **K** (Left) CCK-8 assays measuring cell viability in HDAC2-knockdown HepG2 cells with or without LAPTM4B overexpression. (Right) Cell proliferation curves based on OD values at 450 nm transformed to log2 scale. **L** Subcutaneous xenograft experiment evaluating tumor formation capacity of HDAC2-knockdown HepG2 cells with or without LAPTM4B overexpression (*n* = 7 per group). Scale bar, 5 mm. Tumor volumes were measured. **M** Representative images of IHC staining showing expression levels of HDAC2, LAPTM4B, ATG3, p62, and Ki67 in xenografted tumors formed from HDAC2-knockdown HepG2 cells with or without LAPTM4B overexpression. Scale bar, HE: 100 µm, others: 50 µm. All data were subjected to at least three separate experiments. Data were displayed as mean ± SD. Unpaired Student’s t tests were employed for two-variable comparisons. *p < 0.05; **p < 0.01; ***p < 0.001; ns, no significance in comparison with control group.

Subsequently, we stably transfected HepG2 cells with knockdown or overexpression constructs targeting LAPTM4B, and assessed transfection efficiency using qRT-PCR and western blotting (Fig. S3I–K). The results demonstrated that overexpression of LAPTM4B upregulated the expression of autophagy-related genes ATG3, ATG5, ATG7, Beclin-1, and LC3II, while suppressing the expression of p62 (Fig. 5A, B). Conversely, knockdown of LAPTM4B led to opposite effects (Fig. 5C, D), further validating LAPTM4B’s role in regulating autophagy in HCC. Next, we carefully evaluated the potential impact of LAPTM4B on HCC autophagy modulation by HDAC2. Western blotting and qRT-PCR assays revealed that HDAC2 deficiency reduced the expressions of ATG3, ATG5, ATG7, Beclin-1, and LC3II, and increased p62 expression, whereas restoration of LAPTM4B expression reversed these effects (Fig. 5E, F and Fig. S3N). In addition, IF and TEM assays showed that HDAC2 knockdown resulted in decreased LC3 punctate staining, increased p62 punctate staining and reduced autophagic vesicles, which were rescued by overexpression of LAPTM4B (Fig. 5G–I).

Similarly, we further investigated the role of LAPTM4B in mediating the response to HDAC2 in promoting malignant progression of HCC. HepG2 cells with HDAC2 knockdown exhibited significantly impaired proliferation and clone formation abilities, characterized by a 16.8-h longer cell doubling time and a 78% reduction in clonal clusters. Conversely, re-expression of LAPTM4B restored both growth parameters, with a 26.4-h shorter cell doubling time and a 44% increase in clonal clusters (Fig. 5J, K). To further validate these findings in vivo, we established a xenograft tumor model and observed that the reduced tumor volume in the HDAC2 knockdown group was rescued upon overexpression of LAPTM4B (Fig. 5L). In addition, IHC staining of transplanted tumor tissue sections revealed that both the inhibition of autophagy and suppression of proliferation caused by HDAC2 knockdown were reversed by ectopic expression of LAPTM4B (Fig. 5M). Collectively, these results suggest that HDAC2 promotes autophagy and associated HCC malignancy through upregulation of LAPTM4B.

### HDAC2 enhances autophagy in HCC cells by transcriptionally activating LAPTM4B

**Fig. 6:**
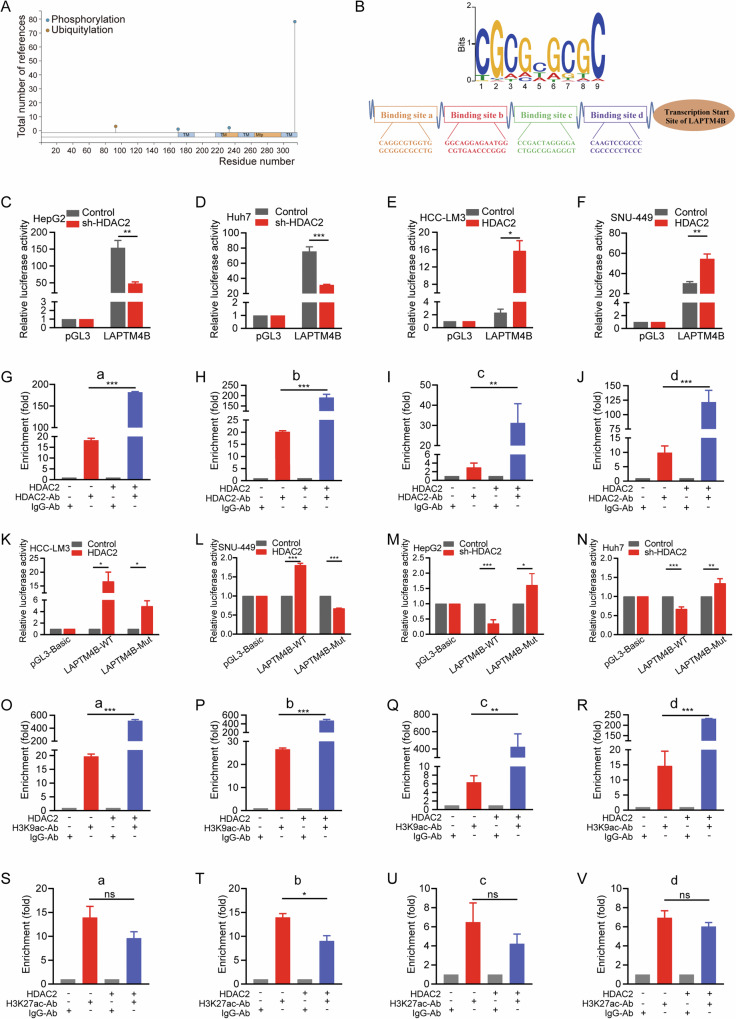
HDAC2 enhances autophagy in HCC cells by transcriptionally activating LAPTM4B. **A** The online database PhosphoSitePlus showing no acetylation modification sites for LAPTM4B. **B** Putative HDAC2-binding sites (HBS) in the promoter sequence of LAPTM4B. **C**, **D** Dual luciferase reporter gene detection was conducted in HepG2 and Huh7 cells with HDAC2-knowndown. **E**, **F** Dual luciferase reporter gene detection was conducted in HCC-LM3 and SNU-449 cells overexpressing HDAC2. **G**–**J** Chromatin immunoprecipitation (ChIP) experiments in HCC-LM3 cells overexpressing HDAC2 to evaluate the binding of HDAC2 to the LAPTM4B promoter. **K**, **L** Dual luciferase reporter gene detection for the mutant sequences of HBS in HCC-LM3 and SNU-449 cells following HDAC2 overexpression. **M**, **N** Dual luciferase reporter gene detection for the mutant sequences of HBS in HepG2 and Huh7 cells knocking down HDAC2. **O**–**R** ChIP assay for H3K9ac was performed in HCC-LM3 cells overexpressing HDAC2 to examine the histone acetylation levels of the four binding sites of HDAC2 and LAPTM4B. **S**–**V** ChIP assay for H3K27ac was performed in HCC-LM3 cells overexpressing HDAC2 to examine the histone acetylation levels of the four binding sites of HDAC2 and LAPTM4B. All data were subjected to at least three separate experiments. Data were displayed as mean ± SD. Unpaired Student’s t tests were employed for two-variable comparisons. *p < 0.05; **p < 0.01; ***p < 0.001; ns, no significance in comparison with control group.

To elucidate the molecular mechanisms by which HDAC2 mediates the regulation of autophagy and autophagy-associated malignant progression in HCC through LAPTM4B, we first investigated the potential post-translational modification [46] mode of LAPTM4B using the PhosphoSitePlus online database. Our analysis revealed no acetylation modification sites for LAPTM4B (Fig. 6A). Given HDAC2’s role as a deacetylase, particularly in regulating gene expression at the transcriptional level, we explored whether HDAC2 promotes the transcriptional activation of LAPTM4B. Utilizing the hTFtarget [47] database, we identified four potential HDAC2-binding sites (HBS) with strong binding capacity in the LAPTM4B gene promoter sequence (Fig. 6B and Fig. S4A). Subsequently, we conducted dual luciferase reporter gene assays, demonstrating that HDAC2 binds directly to the LAPTM4B promoter and enhances its transactivation (Fig. 6C–F). Chromatin immunoprecipitation (ChIP) assays further confirmed this interaction, showing that chromatin fragments containing the identified HBS were significantly enriched by an anti-HDAC2 antibody compared to normal IgG. Moreover, the enrichment efficiency increased with HDAC2 overexpression and decreased with HDAC2 knockdown (Fig. 6G–J and Fig. S4B–F). To ascertain the functional relevance of these HBS, we introduced mutations into the four identified sites and assessed luciferase activity. The results demonstrated that mutations in these HBS significantly reduced luciferase activity induced by HDAC2 (Fig. 6K, L). Similarly, knockdown of HDAC2 markedly decreased luciferase activity, which was restored upon mutation of the HBS (Fig. 6M, N). These findings underscore the critical role of these four HBS in mediating the transcriptional activation of LAPTM4B by HDAC2.

Next, we assessed the histone acetylation status of the identified four HBS on the LAPTM4B promoter using ChIP-qPCR analysis. Our results demonstrated strong enrichment of H3K9ac at these sites. Importantly, HDAC2 overexpression significantly enhanced H3K9ac enrichment (Fig. 6O–R), whereas HDAC2 knockdown led to reduced H3K9ac levels (Fig. S4G–J). Conversely, H3K27ac enrichment was not significantly altered at these binding sites (Fig. 6S–V). These results suggested that HDAC2 may only promote deacetylation of H3K27ac but not H3K9ac and H3K9ac remains at an extremely high level of acetylation, supporting the idea of promoting transcription of downstream genes. Together, these results underscore that HDAC2 binds directly to the LAPTM4B promoter via the four HBS, establishing LAPTM4B as a novel target gene of HDAC2.

### Selective inhibition of HDAC2 attenuates HCC malignant progression

**Fig. 7:**
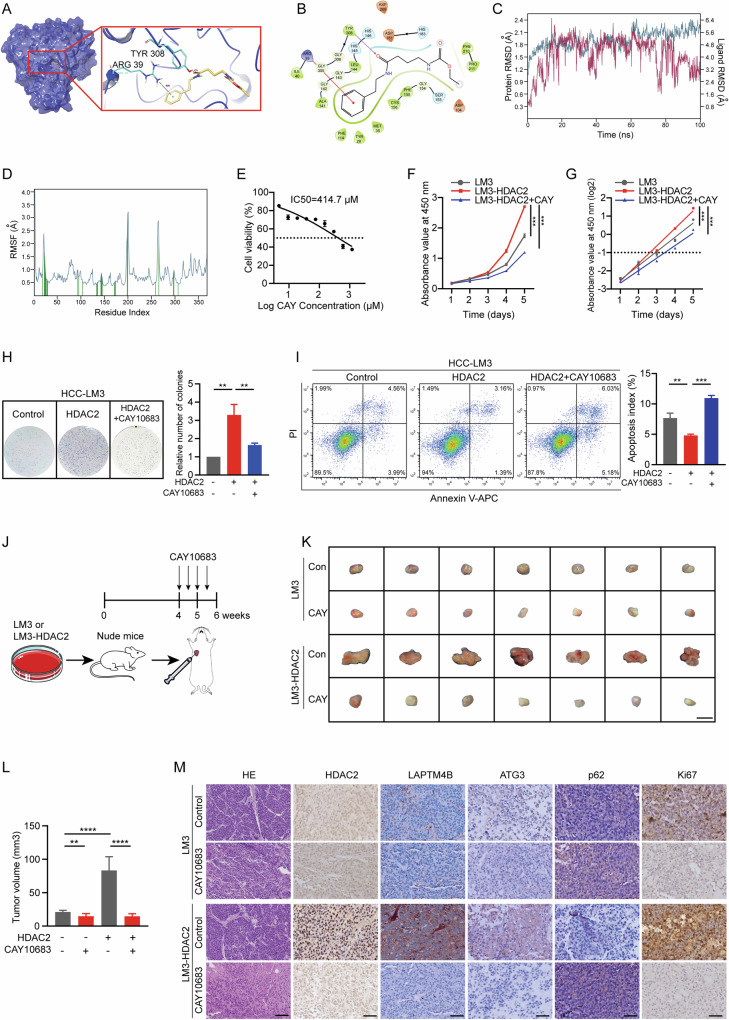
Selective inhibition of HDAC2 attenuates HCC malignant progression. **A** Molecular docking model illustrating CAY10683 binding to HDAC2. **B** 2D representation showing interactions of CAY10683 with specific amino acids, including TYR308 and ARG39. **C** Root mean square deviation (RMSD) values depicting the stability of CAY10683 (red) and HDAC2 (blue) during 100 ns molecular dynamics simulations. **D** Root mean square fluctuation (RMSF) values of HDAC2 residues observed during molecular dynamics simulations. **E** IC50 values of CAY10683 determined by CCK-8 assays in HCC-LM3 cells. **F** CCK-8 assays showing the effect of CAY10683 treatment on cell viability in HDAC2-overexpressing HCC-LM3 cells. **G** Cell proliferation curves based on OD values at 450 nm transformed to log2 scale. **H** (Left) Representative images of colony formation assays in HDAC2-overexpressing HCC-LM3 cells with or without CAY10683 treatment. (Right) Quantification of colony numbers. **I** Cell apoptosis assays in HDAC2-overexpressing HCC-LM3 cells treated with CAY10683. **J** Schematic representation of the subcutaneous tumor model and subsequent intratumoral injection of CAY10683 in nude mice. **K**, **L** Gross images of subcutaneous tumors in nude mice after 4 weeks of growth followed by intratumoral injection of diluted DMSO or CAY10683 (*n* = 7 per group). Scale bar, 5 mm. Tumor volumes were measured. **M** Histological sections (HE: 100 µm, others: 50 µm) of xenograft tumors injected intratumorally with diluted DMSO or CAY10683, showing tumor morphology and treatment effects. All data were subjected to at least three separate experiments. Data were displayed as mean ± SD. Unpaired Student’s t tests were employed for two-variable comparisons. *p < 0.05; **p < 0.01; ***p < 0.001; ****p < 0.0001.

HDAC2 plays a pivotal role in the adverse progression of HCC by transcriptionally activating LAPTM4B, representing a significant therapeutic target. To assess this, we evaluated the effects of Santacruzamate A (CAY10683), a selective HDAC2 inhibitor, on HCC progression. Molecular docking simulations demonstrated direct interaction between CAY10683 and HDAC2 (Fig. 7A, B), further confirmed by molecular dynamics simulations showing stable conformational binding (Fig. 7C, D). CCK8 assay revealed that HDAC2 overexpression in HCC-LM3 cells accelerated proliferation, reducing doubling time by 7.2 h, whereas CAY10683 treatment significantly inhibited proliferation, prolonging doubling time by 19.2 h (Fig. 7E–G). In addition, CAY10683 markedly reversed HDAC2-induced clone formation ability, decreasing it by 50% (Fig. 7H). Flow cytometry showed that CAY10683 enhanced apoptosis by 1.3-fold compared to HDAC2-induced inhibition (Fig. 7I). In vivo studies using subcutaneous injection models in nude mice confirmed the inhibitory effect of CAY10683 on HCC progression. Four treatments with CAY10683 reduced tumor volume by 31% in LM3 + CAY10683 group and 82% in LM3-HDAC2 + CAY10683 group after four weeks (Fig. 7J–L). IHC staining of tumor tissues demonstrated that CAY10683 effectively suppressed HDAC2 expression, attenuating both HDAC2-induced autophagy and proliferation, as well as LAPTM4B expression (Fig. 7M). HE staining of major organs in tumor-bearing mice revealed that CAY10683 had no significant side effects on mice (Fig. S5A). These findings underscore the potent inhibitory effect of the specific HDAC2 inhibitor on HCC malignancy, highlighting its therapeutic potential in targeting HDAC2-mediated pathways.

### HDAC2 and LAPTM4B are clinically relevant and predict adverse outcomes in HCC patients

**Fig. 8:**
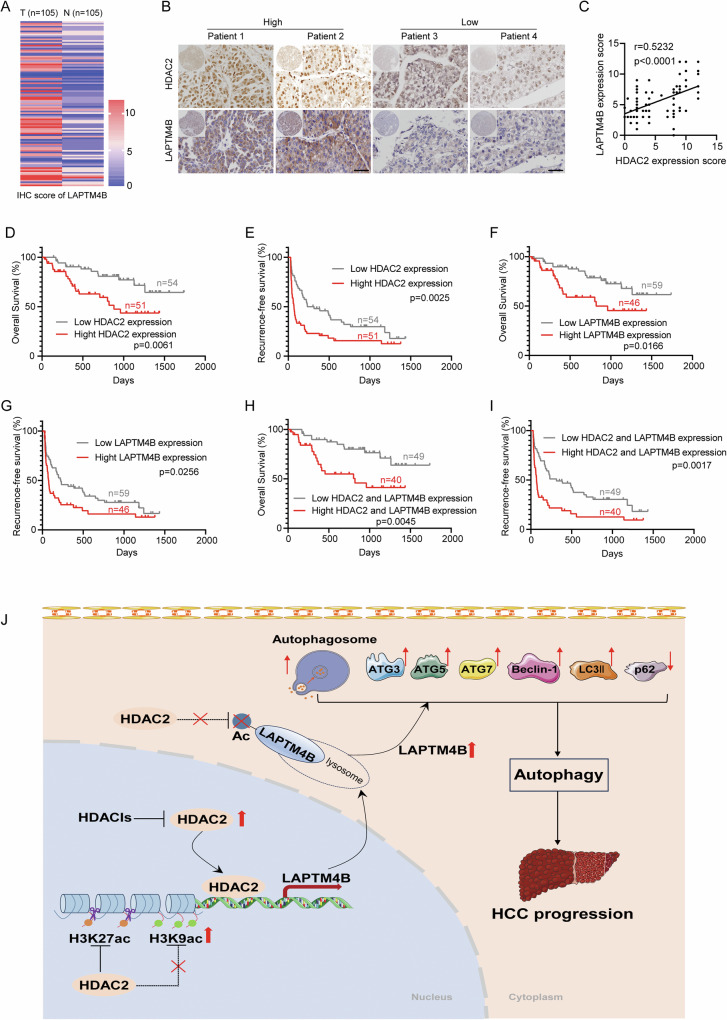
Pathological association of HDAC2 with LAPTM4B in HCC. **A** The IHC score of LAPTM4B in HCC TMAs. **B** Representative images of HDAC2 and LAPTM4B staining on HCC TMAs. Scale bar, 50 µm. **C** Pearson’s correlation analysis was utilized to assess the association between HDAC2 and LAPTM4B in 105 HCC individuals. **D**, **E** KMSA of OS and recurrence-free survival (RFS) in HCC patients based on HDAC2 expression in HCC TMAs (log-rank test). **F**, **G** KMSA of OS and RFS in HCC patients based on LAPTM4B expression in HCC TMAs (log-rank test). **H**, **I** KMSA of OS and RFS in HCC patients based on HDAC2 and LAPTM4B expression in HCC TMAs (log-rank test). **J** Schematic model of the mechanism by which HDAC2 directly binds LAPTM4B and facilitates its transcriptional activation, which in turn promotes autophagy and autophagy-related malignant progression in HCC. Selective HDAC2 inhibitors (HDACIs) reverse these effects. Data were displayed as mean ± SD. Unpaired Student’s t tests were employed for two-variable comparisons.

The preceding findings clearly establish the pivotal role of LAPTM4B in HDAC2-mediated pro-autophagy and tumorigenicity. We further investigated the expression and connection of HDAC2 and LAPTM4B in surgical specimens of HCC, along with their impact on clinical prognosis. As anticipated, IHC staining of TMAs containing 105 HCC specimens revealed markedly higher expression levels of HDAC2 and LAPTM4B in HCC tissues compared to adjacent normal tissues (Figs. 1J and 8A). Further analysis underscored a strong co-expression pattern between HDAC2 and LAPTM4B, showing a high positive correlation (Fig. 8B, C and Table S1).

Furthermore, we conducted an analysis of clinical follow-up records from 105 HCC patients to explore the correlation between HDAC2 expression and clinicopathological features, including tumor sizes, cirrhosis, HBV infection, and microvascular invasion. Our findings revealed that high HDAC2 expression significantly correlated with microvascular invasion (*p* = 0.004) and Edmondson classification (*p* = 0.007) (Table S2).

Subsequently, we employed Kaplan–Meier analysis to assess the association of HDAC2 and LAPTM4B expression levels with patient outcomes in HCC. Elevated levels of HDAC2 and LAPTM4B were notably associated with poorer prognosis, characterized by shorter overall survival (OS) and recurrence-free survival (RFS) (Fig. 8D–G). Importantly, patients exhibiting high expression of both HDAC2 and LAPTM4B showed even shorter OS and RFS compared to those with low expression of both markers (Fig. 8H, I). These results underscore that increased HDAC2 and LAPTM4B expression may serve as indicators of unfavorable prognosis in HCC patients. Moreover, HDAC2 expression emerged as an independent risk factor for HCC patients according to both univariate and multivariate Cox risk analyses (Table 1). In conclusion, our findings confirm elevated HDAC2 and LAPTM4B expression in HCC, and their association with poor clinical outcomes, supporting the hypothesis that HDAC2 promotes HCC autophagy and malignant progression by activating LAPTM4B transcription.

**Table 1:** Univariate and multivariate assays of factors related to overall survival in HCC patients (*n* = 105).

Clinical variables	Univariate analysis			Multivariate analysis		
	HR	95%CI	*p* value	HR	95%CI	*p* value
Sex (Male versus Female)	3.317	0.792–13.892	0.101			
Age (>60 versus ≤60 years)	0.440	0.198–0.977	0.044			
Tumor size (≥10 versus <10 cm)	1.844	0.925–3.676	0.082			
Cirrhosis (Yes versus No)	3.139	1.295–7.606	0.011*	1.804	0.881–3.696	0.107
Microvascular invasion (Yes versus No)	3.302	1.004–10.855	0.049*	1.226	0.392–3.834	0.726
Microsatellites (Yes versus No)	3.259	1.572–6.753	0.001**	1.576	0.834–2.978	0.161
HBsAg (Yes versus No)	3.463	1.334–8.987	0.011*	2.209	1.071–4.554	0.032*
Edmondson classification (III/IV versus I/II)	6.674	2.029–21.951	0.002**	2.477	0.985–6.227	0.054
HDAC2 expression	1.304	1.122–1.516	0.001**	1.433	1.238–1.659	<0.001***

Statistically significant; *p < 0.05; **p < 0.01; ***p < 0.001.

## Discussion

HCC remains a significant global health challenge [48]. Increasing evidence underscores the critical role of autophagy in the HCC malignant progression [49–51]. However, the precise upstream regulatory network of autophagy and its specific mechanisms driving the malignant advancement of HCC remain poorly understood. Unraveling these mechanisms could pave the way for innovative HCC therapies.

Previous studies have indeed highlighted HDAC2 as a potential oncogene involved in the malignant progression of HCC, contributing to poor patient outcomes [9, 52, 53]. However, the specific mechanisms underlying its oncogenic role, particularly its relationship with autophagy, have not been fully elucidated in the field of oncology until now. In our study, we identified HDAC2 as a crucial regulator capable of modulating autophagy in HCC through a combination of database screening, in vitro experiments, and in vivo validations. Our findings provide compelling evidence that HDAC2 is significantly upregulated in HCC tissues and is associated with poor prognosis. Importantly, we demonstrated that HDAC2 promotes autophagy in HCC cells, thereby enhancing the malignant progression of the disease. This research significantly advances our understanding of HDAC2’s function in HCC progression, shedding light on its mechanistic involvement in autophagy regulation. These insights not only deepen our knowledge of cancer pathophysiology but also open avenues for exploring personalized therapeutic strategies targeting HDAC2 in HCC.

Considering that lysosomes are crucial executors of autophagy function [54], and based on RNA-seq data, we focused on lysosome-related genes as downstream targets of HDAC2. It has been shown that LAPTM4B takes a significant role in autophagy regulation in cancers [24, 25, 55]. In our study, we confirmed that HDAC2 regulates LAPTM4B to promote autophagy, leading to malignant progression of HCC. The underlying mechanism was next further investigated. Since HDAC2’s deacetylation function, we first excluded the possibility that HDAC2 regulates post-translational modifications of LAPTM4B by database prediction. Based on the fact that HDAC2 can largely regulate the mRNA level of LAPTM4B, we confirmed that HDAC2 binds to LAPTM4B through four specific HBS and promotes its transcriptional activation. By mutating these four HBS, the transcriptional activation of LAPTM4B by HDAC2 is greatly diminished.

Previous studies have established HDAC2’s role in deacetylating the N-termini of histone H3 and H4, leading to a compact nucleosome structure and transcriptional repression of downstream genes [10, 56]. However, our study diverges from this norm by revealing that HDAC2 actually promotes the transcriptional activation of LAPTM4B, necessitating a thorough investigation into its underlying mechanism. We found that the acetylation level of H3K9ac was elevated at these four sites, with a pronounced increase observed upon HDAC2 overexpression and a decrease upon HDAC2 knockdown. In contrast, H3K27ac enrichment at these sites was not significant, suggesting that HDAC2 predominantly deacetylates H3K27ac but not H3K9ac. Despite HDAC2’s classification as a deacetylase, the absence of deacetylation at H3K9ac and the absence of acetylation modification sites in LAPTM4B indicate that HDAC2’s binding elevates LAPTM4B’s overall acetylation levels. This in turn increases chromatin accessibility and maintains LAPTM4B in a transcriptionally active state, underscoring the direct role of HDAC2 in promoting LAPTM4B transcriptional activation. These findings collectively highlight LAPTM4B as a unique and direct target gene of HDAC2.

Based on our findings linking high HDAC2 expression to adverse outcomes in HCC patients and identifying it as an independent risk factor for HCC, targeting HDAC2 with inhibitors may enhance anticancer strategies by suppressing cancer cell growth and autophagy. Encouragingly, multiple HDAC inhibitors (HDACIs) have been explored for their antiproliferative effects across various cancers [57]. For instance, Deubzer et al. demonstrated that HC toxin, an HDAC inhibitor, effectively inhibited migration and invasion in MYCN-amplified neuroblastoma cells [58]. Furthermore, several HDACIs have shown promising activity in clinical trials against cancers such as T-cell lymphoma and metastatic renal cancer [59]. To validate our findings, we conducted experiments using the highly specific HDAC2 inhibitor CAY10683 at both cellular and animal levels. Our results indicate that inhibition of HDAC2 expression significantly mitigates the malignant behavior of HCC and suppresses tumor growth, along with reduced autophagy levels. In summary, our observations underscore the potential of HDAC2 and its target gene LAPTM4B as prognostic indicators and promising therapeutic targets for HCC. However, further investigation through extensive clinical trials is warranted to determine their efficacy in clinical settings.

Collectively, our study demonstrated that HDAC2 expression was markedly upregulated in HCC tissues and correlated with a poorer prognosis, independently predicting outcomes for HCC patients. In addition, HDAC2 directly binds to and activates the transcription of LAPTM4B, thereby enhancing autophagy in HCC and promoting its malignant progression (Fig. 8J). Critically, targeted inhibition of HDAC2 expression significantly reversed the adverse progression of HCC (Fig. 8J). Interestingly, while prior studies have emphasized HDAC2’s role in deacetylating histones and repressing gene transcription, our study highlights its direct involvement in promoting LAPTM4B transcription, identifying LAPTM4B as a novel direct target gene of HDAC2. These findings highlight HDAC2 as a key regulator in HCC, shedding light on its role in promoting autophagy and driving malignant progression through LAPTM4B activation. Overall, the frequent aberrant activation of the HDAC2-LAPTM4B axis contributes to increased autophagy levels in HCC and correlates with a poorer prognosis. Thereby, targeting LAPTM4B may also improve the antitumor effects in HCC by inhibiting autophagy or antagonize HDAC2 dysregulated tumors, which requires subsequent more specific studies to determine. Considering the promising antitumor therapeutic strategy for inhibiting autophagy, in future studies, we can combine inhibition of the HDAC2-LAPTM4B axis with other modes of autophagy regulation to explore whether combination therapy can achieve synergistic and toxicity-reducing effects. Altogether, our study enhances understanding of HDAC2’s role in autophagy regulation and malignant progression in HCC, underscores the therapeutic potential of targeting HDAC2 in HCC treatment, offering a promising target for personalized therapeutic strategies.

## Materials and methods

### Tissue specimen collection

The paraffin-embedded tissue samples utilized in our research were obtained from patients operated for HCC by hepatectomy between May 2015 and December 2018 at Mengchao Hepatobiliary Hospital of Fujian Medical University. These patients had not received any anticancer treatment prior to surgery and had comprehensive clinicopathologic information and follow-up records available. All specimens were pathologically confirmed as HCC. The sample collection, clinicopathologic information acquisition and patient follow-up involved in the study were in accordance with the requirements of the Ethics Committee of Fujian Medical University. Patients were aware of the content of the study and voluntarily signed an informed permission before collecting information and samples.

### Cell line and culture

The human hepatoma cell lines HepG2 and Huh7 were purchased from Procell (Wuhan, China) and the National Collection of Authenticated Cell Cultures (Shanghai, China). HCC-LM3 and SNU-449 were obtained from iCell Bioscience Inc (Shanghai, China) and Immocell Biotechnology Co., Ltd (Xiamen, China). Cells were identified by short tandem repeat (STR) sequence analysis and tested negative for mycoplasma contamination by PCR-based detection. HepG2 cells were grown in Minimum Essential Medium (MEM, Procell, Wuhan, China), Huh7 and HCC-LM3 cells were incubated in Dulbecco’s modified Eagle’s medium (DMEM, HyClone, Logan, UT, USA), and SNU-449 were maintained in RPMI 1640 medium (HyClone, Logan, UT, USA). All medium were supplemented with 10% FBS (Gibco, USA) and 1% Penicillin-Streptomycin (100 U; Gibco), and the cells were cultured in an incubator at 37 °C with 5% CO_2_.

### Cell transfection and establishment of stably transduced cell lines

HDAC2 plasmid, vectors harboring HDAC2 small hairpin RNAs (shRNA) and the corresponding control vector were obtained from Hanbio Technology (Shanghai, Chaina). LAPTM4B plasmid and shRNA vector have been manufactured and stored in our laboratory. The promoter sequence of LAPTM4B was chemically synthesized by Tsingke Biotechnology (Beijing, China), which ends with KpnI and BglII recognition sites. It was then cloned into the pGL3-Basic vector (Promega, Madison, WI, USA) to generate the corresponding luciferase reporter plasmids. Similarly, new sequences were synthesized by mutating the four potential binding sites of HDAC2 and LAPTM4B to construct the LAPTM4B promoter mutant plasmid (OBiO Technology, Shanghai, China). Transfection was conducted using Lipofectamine 3000 transfection reagent (Lipo 3000, Invitrogen, Carlsbad, CA, USA) according to the manufacturer’s instructions. 3 × 10^5^ cells were seed into six-well plates and cultured overnight. The ratio of DNA plasmid to Lipo 3000 was 1:1.5. Specifically, Opti-MEM (Gibco, USA) was used to dilute Lipo 3000, DNA plasmid and P3000, respectively. After gentle mixing and incubation for 15 min, the mixture was added to the cells for transfection and incubated for 8 h. Finally, cells were replaced with fresh medium and continued to be cultured for 48–72 h to assess transfection efficiency.

Lentiviral packaging was performed in order to generate cell lines with stable overexpression or knockdown of HDAC2 or LAPTM4B. Briefly, 293T cells were cotransfected at 50% confluence with the corresponding plasmids and lentiviral packaging plasmids REV, RRE, and VSVG (Invitrogen, Carlsbad, CA, USA). The culture medium was collected at 48 h and 72 h and filtered by a 0.45 μm filter, and then added to the plated HCC cells that had been inoculated in six-well plates with 1 × 10^5^ cells per well. After 48 h of culture, the cells were continuously screened with 1 µg/mL puromycin (Beyotime Biotech, Shanghai, China) or 300 µg/mL of G418 (Sigma-Aldrich, St. Louis, MO, USA) for 7–14 days. Cells with stable overexpressing or knockdown of HDAC2 or LAPTM4B were used for further studies.

### Tissue microarrays (TMAs) and immunohistochemical (IHC) staining

Based on the above screening criteria, 105 HCC tumor tissues and paired normal liver tissues were used to produce TMAs. For IHC, tissue sections were rehydrated with gradient-reduced ethanol after being deparaffinized with xylene. Endogenous catalase was blocked with 3% hydrogen peroxide followed by antigen repair in Tris-EDTA or 10 mM sodium citrate buffer (pH 6.0). After 5% BSA occlusion, the primary antibody was incubated at 4 °C overnight. Histochemistry secondary antibodies were incubated for 1 h and color visualized using DAB and restained with hematoxylin as well as differentiated by hydrochloric acid alcohol. Neutral gum sealed the slices and then observed under the microscope.

Staining results were analyzed independently by two pathologists who were uninformed about the patients. A 4-point scale was used to rate the intensity of cellular staining: 0 represented no positive staining, 1 light yellow, 2 yellow-brown, and 3 brown. The percentage of positive cells was graded from 0 to 4, as 0 (<5%), 1 (5–25%), 2 (26–50%), 3 (51–75%), and 4 (76–100%). The final score was then calculated by multiplying the two values.

### Immunofluorescent (IF) staining

The cells were cultured at a density of 2 × 10^3^ cells/well in 24-well culture plates, and fixed for 30 min with 4% paraformaldehyde at room temperature, and then the membranes were broken for 15 min with 0.2% Triton X-100. After being blocked for 1 h in 5% fetal bovine serum, the cells were treated overnight at 4 °C with the corresponding primary antibody. Upon washing three times with PBST, cells were incubated with fluorescent secondary antibody (Goat anti-rabbit IgG-Alexa Fluor 594-conjugated (ab150080, red) or Goat anti-rabbit IgG-Alexa Fluor 488-conjugated (ab150077, green)), followed by nuclear staining with 4’,6-diimino-2’-phenelindole dihydrochloride (DAPI, AR1176). Finally, a confocal microscope (Olympus FV1000, Japan) was used to capture IF images.

### Transmission electron microscopy (TEM)

Approximately 5 × 10^6^ cultured cells were digested and centrifuged (1000 rpm, 8 min) to obtain samples. Cell precipitation blocks were first fixed for several days at 4 °C with 3% glutaraldehyde-1.5% paraformaldehyde, then exchanged for 1% osmium-1.5% potassium ferrocyanide for 1.5 h, and repeatedly washed with PBS. The sections were further stained with 70% alcohol-saturated uranyl acetate dye, dehydrated with alcohol-acetone gradient, and embedded with epoxy resin 618 embedding agent. 90 nm Ultrathin slices were stained for 5–15 min each with uranyl acetate and lead citrate, and then observed and captured under a TECNAI-type transmission electron microscope from FEI.

### Cell proliferation assay and colony formation assay

HCC cells were inoculated in 96-well plates at a density of 1 × 10^3^ and cell proliferation was evaluated using the CCK-8 (C6005, US Everbright, Inc.) following the production description. After adding CCK8 solution, the cells were incubated for 1 h. The absorbance OD value of the cells at 450 nm was measured on microplate readers. Measurements were taken continuously for 5 days, and cell growth curve was plotted. In the colony formation test, 1 × 10^3^ HCC cells were cultured in 6-well plates continuously for two weeks until colonies consisting of ≥50 cells were formed as visible to the naked eye. After being fixed for 30 min with paraformaldehyde, the colonies were stained for 1 h with crystal violet dye (Beyotime Biotech, Shanghai, China) and thereafter counted.

### Animal experiments

3–5-week-old male BALB/c nude mice were obtained from Guangdong GemPharmatech Co., Ltd. The mice were housed in an SPF-grade barrier in the animal rooms of Fujian Medical University. All animal experimental groups were randomized with a minimum of seven animals per group. In the nude subcutaneous tumorigenesis model, 100 μL of HCC cell suspension (2 × 10^6^ cells) was subcutaneously injected into the axilla of nude mice. Measurement of the tumor length (*L*) and width (*W*) was performed every 4 days. Tumor volume was calculated as follows: volume (mm^3^) = (*W*^2^ × *L*)/2. The mice were executed after four weeks, and the tumors tissues were stripped, fixed and embedded in paraffin for further examination. All animal experiments were conducted in strict conformance with the National Institutes of Health Guidelines for the Care and Use of Animals and approvable by the Animal Care and Use Committee of Fujian Medical University.

Four weeks following the subcutaneous injection of LM3 or LM3-HDAC2 cells, the subcutaneous tumorigenesis model was successfully established in nude mice. Subsequently, the nude mice were treated with intratumoral injections of the HDAC2 inhibitor Santacruzamate A (CAY10683, Selleck, Shanghai, China). The drug was prepared at a dose of 10 mg/kg according to the manufacturer’s instructions. To administer the drug, the skin of the nude mice was disinfected first. Then, a needle was inserted ~1 cm subcutaneously at the outer edge of the tumor, advanced to the center of the tumor subcutaneously, withdrawn without blood, and the drug was injected slowly and uniformly.

### RNA Sequencing

Three replicates of HepG2 and HepG2 sh-HDAC2 cell samples were collected, and total RNA was extracted by adding appropriate amount of TRIzol reagent (Invitrogen, Carlsbad, CA, USA). After quality control of RNA, sequencing library production was carried out by using the stranded mRNA library production kit (Wuhan Seqhealth Co., Ltd. China). Sequencing was undertaken by Wuhan Seqhealth Co., Ltd on the Illumina Hiseq platform (Project No: KC2023-H1612). Using the edgeR package (version 3.12.1), the obtained raw data were further processed for visualization and screening of differentially expressed genes (DEGs) in the two groups. The screening conditions were absolute value of log fold-change > 1 and *p* value < 0.05. The final DEGs were performed Gene Ontology (GO) enrichment analysis, Kyoto Encyclopedia of Genes and Genomes (KEGG) pathway analysis and other bioinformatic analyses for downstream analysis and data mining.

### Dual luciferase reporter gene assay

HCC cells were inoculated into 24-well plates at 8 × 10^4^ density and cultured until 80% confluent. Then pGL3-basic-LAPTM4B promoter-luciferase reporter or pGL3-basic-LAPTM4B promoter mutation-luciferase reporter along with pRL-TK (Promega) were transfected into the above cells. After 48 h of incubation, Dual luciferase reporter gene assay (Promega, Madison, WI, USA) was executed and the procedure described by the manufacturer was followed. The firefly luciferase activity was normalized to Renilla luciferase activity to obtain relative luciferase activity (RLA). Finally, the differences in RLA between HCC cells with HDAC2 overexpression or knockdown and normal cells were compared.

### Chromatin immunoprecipitation (ChIP) assay

HCC cells were cross-linked for 10 min with freshly produced formaldehyde (final concentration 1%) at room temperature. Then, 125 mM glycine was added for 5 min to terminate the reaction. After two washes with cold PBS, the cells were scratched and centrifuged to collect the precipitate. Subsequently, cells were lysed with ChIP lysis buffer (10 mM Tris-HCl, pH 7.5; 10 mM NaCl; 0.5% NP-40) on ice and chromatin DNA was sonicated (80% power, 20 s on, 40 s off, 15 cycles) to obtain a fragment of ~500 bp. 2% of the supernatant was obtained as Input and the remaining supernatants were incubated with IgG (30000-0-AP, Proteintech), HDAC2 (#57156, CST), H3K9ac (#61252, Active Motif) or H3K27ac (#39134, Active Motif) antibodies overnight at 4 °C with rotation. Next, antibodies-bound chromatin was precipitated with pre-washed ChIP-Grade Protein G Magnetic Beads (#9006, CST) and continued incubation for 3 h at 4 °C. DNA was harvested utilizing the DNA Purification and Recovery Kit (DP214, Tiangen) after elution of the magnetic beads and de-cross-linking of the protein-DNA complexes. Finally, qPCR analysis was performed to identify putative HDAC2-binding sites in the LAPTM4B promoter region and the level of acetylation at these sites.

### Molecular docking and molecular dynamics simulations

Molecular docking simulations were conducted using Schrödinger software. Initially, the protein structure of the target gene HDAC2 (UniProt accession: Q92769) was obtained from the Uniprot database (https://www.uniprot.org) and optimized. A corresponding receptor grid was generated using the software. Subsequently, the structure of Santacruzamate A (CAY10683) was drawn using the 2D Sketcher and optimized for docking using LigPrep with default parameters. Ligand docking was performed in standard precision (SP Docking) mode using Glide, with other parameters set to default. The molecular docking results yielded compound-protein complexes, which were analyzed using Maestro.

Molecular dynamics (MD) simulations were carried out using the Dynamics module (Desmond v53011) within Schrödinger software. Initially, the complexes were prepared using the Protein Preparation module with default parameters. The protein structures were minimized and optimized, and then imported into the System Builder module. Solubilization was achieved using the TIP3P water model and the OPLS3 force field, followed by neutralization with Na+. MD simulations were conducted over 100 ns after energy minimization and system equilibration. Finally, the Simulation Interactions Diagram function was utilized to analyze the output files from molecular dynamics simulations. The root mean square deviation (RMSD) was employed to quantify the average displacement of selected atoms relative to a reference frame.

### Statistical analysis

GraphPad Prism 8 (GraphPad, USA) and SPSS software (SPSS22.0; SPSS INC., Chicago, IL, USA) were used for the statistical analyses. Applying the student’s t-test, the differences among the two groups were assessed. Pearson chi-square test was employed to examine the association between HDAC2 expression and clinical characteristics in HCC tissues, and the correlation between HDAC2 and LAPTM4B expression. Survival analysis was performed by Kaplan–Meier assay with log-rank test employed to verify the significance. To determine whether HDAC2 was a separate prognostic indicator for HCC, univariate and multivariate Cox regression analysis were used. All experiments were performed independently at least 3 times, with 3 technical replications. Data were expressed as mean ± standard deviation (SD) and *p* < 0.05 was regarded statistically significant.

### Supplementary information

Supplementary information for HDAC2 promotes autophagy-associated HCC malignant progression by transcriptionally activating LAPTM4B

Original western blots for HDAC2 promotes autophagy-associated HCC malignant progression by transcriptionally activating LAPTM4B

## Data Availability

Data and materials are available by contacting the corresponding author for reasonable requests.
