# Supplementary material for: HDAC2 promotes autophagy-associated HCC malignant progression by transcriptionally activating LAPTM4B
Source: Cell Death Dis. 2024 Aug 15;15(8):593. doi: 10.1038/s41419-024-06981-3 (PMC11327261; doi:10.1038/s41419-024-06981-3)
Supplement: Supplementary file 1 — Supplementary information for HDAC2 promotes autophagy-associated HCC malignant progression by transcriptionally activating LAPTM4B [file 41419_2024_6981_MOESM1_ESM.docx]

**Supplementary information for**

**HDAC2 promotes autophagy-associated HCC malignant progression by transcriptionally activating LAPTM4B**

Meifeng Wang^#1 2^, Jianping Liao^#1 2^, Jie Wang^#1 2^, Meifang Xu^1 2^, Ye Cheng^1 2^, Lixin Wei^3^*, Aimin Huang^1 2^*

**This file includes：**

Supplementary Materials and Methods

Supplementary Figure S1 to S5

Supplementary Table S1 to S4

**Supplementary Materials and Methods**

**Quantitative real-time polymerase chain reaction (qRT-PCR)**

PrimeScript RT reagent Kit (Takara Bio, Tokyo, Japan) was used to reverse transcribe total RNA, which were isolated from HCC cells and frozen tissues using TRIzol reagent (Invitrogen, Carlsbad, CA, USA). qRT-PCR was conducted with the TB Green Premix Ex Taq (Takara Bio, Tokyo, Japan) according to the manufacturer's guidelines on the CFX Real-Time PCR Detection System (Bio-Rad, Hercules,CA, USA). The 18S gene was used as the reference gene when calculating the relative mRNA levels using the 2^-ΔΔCt^ method. All primers utilized in this experiment are shown in Table S3.

**Western blotting (WB)**

Cells/tissues were harvested into RIPA lysis buffer (Beyotime Biotech, Shanghai, China) comprising protease inhibitors (Beyotime Biotech) for lysis. Protein samples were electrophoresed on 10% or 12% SDS/polyacrylamide gels, and then were moved to polyvinylidene difluoride (PVDF) membranes (Millipore, USA). After blocking the membranes with 5% milk for 1.5 hours at room temperature, the primary antibody was applied overnight at 4 °C. The membranes were then treated for 1.5 hours at room temperature with peroxidase-conjugated HRP-coupled secondary antibody (BA1054, Boster; #31430, Invitrogen). β-Actin (#4970, Cell Signaling Technology) was used as an internal reference protein control. Finally, enhanced BeyoECL Star (Beyotime Biotechnology) was used to visualize the proteins. Antibodies used are shown in Supplementary Table S4.

**Flow cytometry**

Utilizing an Annexin V-APC / PI apoptosis detection kit (Procell, P-CA-207) following the manufacturer's guidelines, flow cytometry was used to determine the apoptosis rate of HCC cells. Briefly, 5 × 10^5^ resuspended HCC cells were harvested, centrifuged for 5 min at 300 g, and then twice rinsed with PBS. After adding 5 µL of Annexin V-APC staining solution and 5 µL of PI staining solution (50 µg/mL), the cells were cultured at room temperature in the dark for 15-20 minutes. Approximately 1 x 10^4^ cells were then collected through Beckman-Coulter XL flow cytometer and FlowJo software (Treestar, USA) was used for analysis. The combined percentage of early (Annexin V+, PI-) and late (Annexin V+, PI-) apoptotic cells was the proportion of apoptotic cells.

**Supplementary Figures and Legends**

**
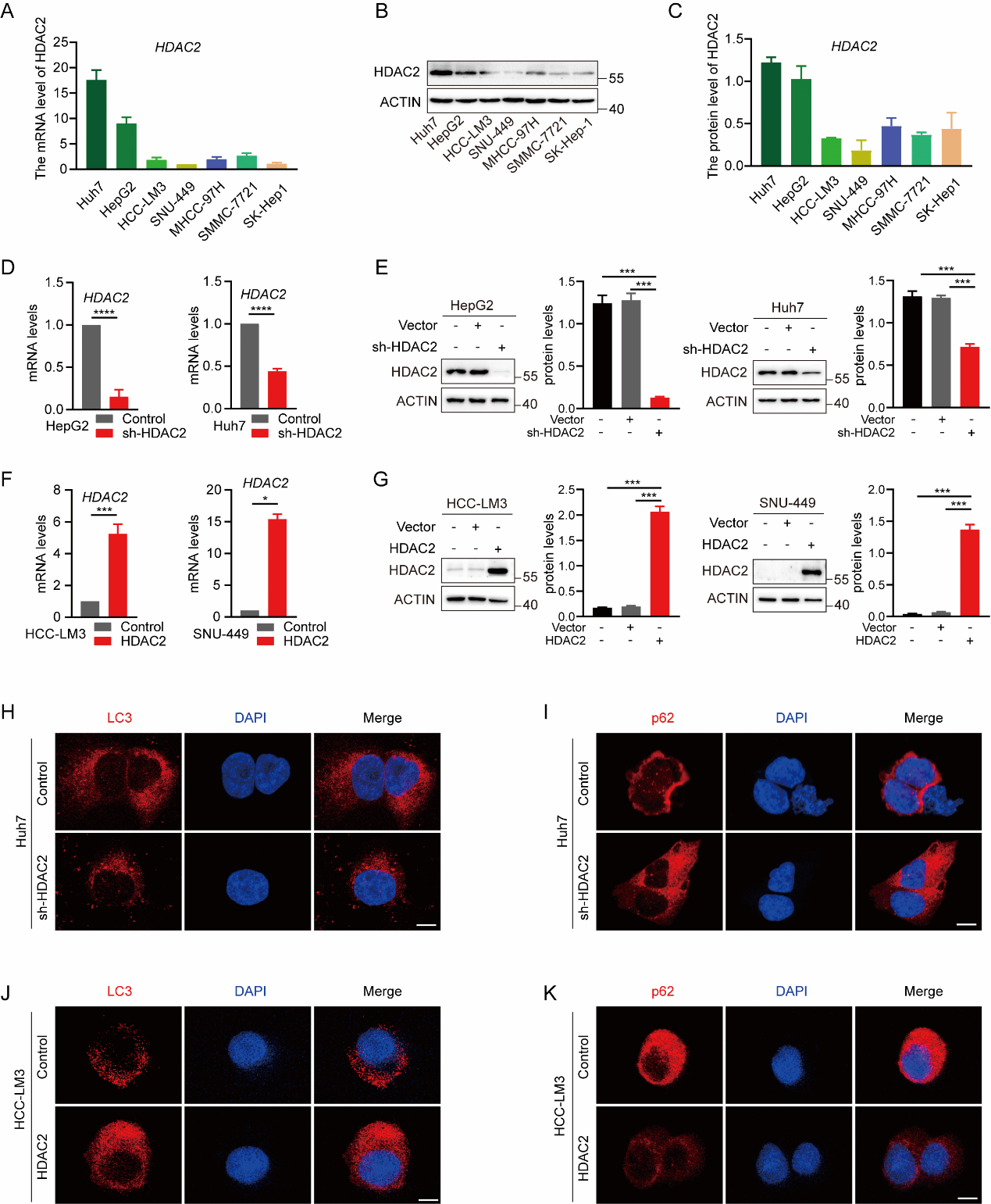
**

**Figure S1. HDAC2 regulates autophagy in HCC tumor cells, Related to Figure 2**

(**A**) (**B**) (**C**) The mRNA and protein level of HDAC2 in seven HCC cell lines (Huh7, HepG2, HCC-LM3, SNU-449, MHCC-97H, SMMC-7721, and SK-Hep1) were detected by qRT-PCR and western blotting. (**D**) (**E**) Detection of the knockdown efficiency of HDAC2 in HepG2 and Huh7 cells. (**F**) (**G**) Examining the overexpression efficiency of HDAC2 in HCC-LM3 and SNU-449 cells. (**H**) (**I**) Immunofluorescence (IF) staining of LC3 and p62 in Huh7 cells with HDAC2 knockdown or not. Red puncta signify LC3 or p62 and blue puncta signify DAPI. Scale bar, 50 µm. (**J**) (**K**) IF staining of LC3 and p62 in control cells and HDAC2 overexpressed HCC-LM3 cells. Red puncta signify LC3 or p62 and blue puncta signify DAPI. Scale bar, 50 µm. All data were subjected to at least three separate experiments. Data were displayed as *mean ± SD*. *Unpaired Student's t tests* were employed for two-variable comparisons. **, p< 0.05; **, p< 0.01; ***, p< 0.001; ****, p< 0.0001*; ns, no significance in comparison with control group.

**
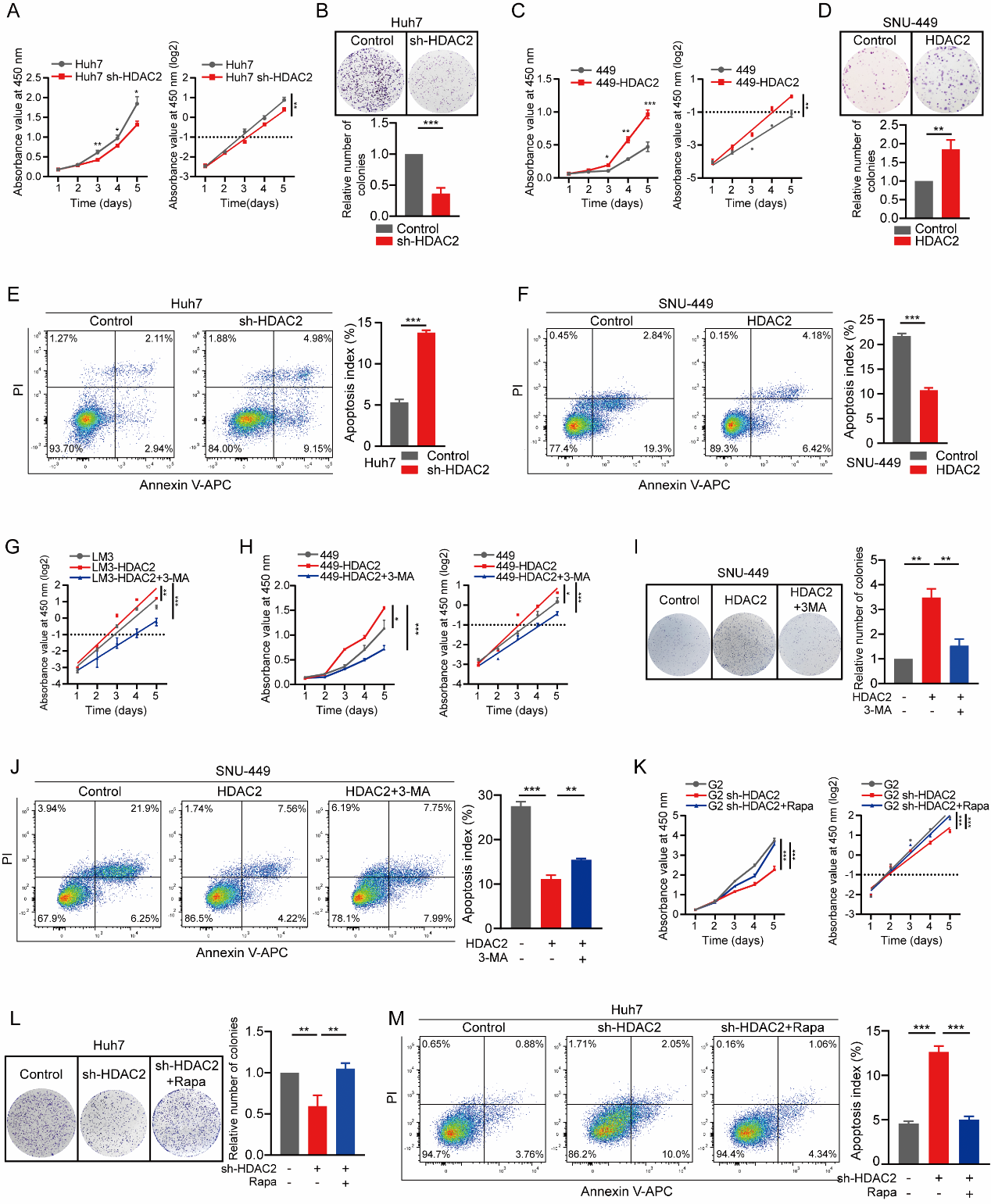
**

**Figure S2. HDAC2 promotes autophagy-associated HCC malignancy, Related to Figure 3**

(**A**) (**C**) (Left) CCK-8 assays measuring cell viability in Huh7 and SNU-449 cells with HDAC2 knockdown or overexpression, respectively. (Right) Cell proliferation curves based on OD values at 450 nm transformed to log2 scale. (**B**) (**D**) (Top) Representative colony formation photos of HCC cells with HDAC2 knockdown or overexpression. (Bottom) Quantification of colonies formed. (**E**) (**F**) Cell apoptosis assays in Huh7 and SNU-449 cells with HDAC2 knockdown or overexpression, respectively. (**G**) Cell proliferation curves of HCC-LM3 cells with HDAC2 overexpression based on OD values at 450 nm transformed to log2 scale. The autophagy inhibitor 3-MA was employed to perform salvage assays. (**H**) (Left) CCK-8 assays in SNU-449 cells with or without HDAC2 overexpression. (Right) Cell proliferation curves based on OD values at 450 nm transformed to log2 scale. The autophagy inhibitor 3-MA was employed to perform salvage assays. (**I**) (Left) Representative colony formation images of HDAC2-overexpression in SNU-449 cells. 3-MA was employed as an autophagy inhibitor. (Right) Quantification of colonies formed. (**J**) Cell apoptosis assays in SNU-449 cells with HDAC2-overexpression. 3-MA was employed as an autophagy inhibitor. (**K**) (Left) CCK-8 assays in HepG2 cells with or without HDAC2 knockdown. (Right) Cell proliferation curves based on OD values at 450 nm transformed to log2 scale. Rapa was used as an autophagy agonist. (**L**) Representative colony formation images of Huh7 cells with HDAC2 knockdown. Rapa was used as an autophagy agonist. (**M**) Cell apoptosis assays in Huh7 cells with HDAC2 knockdown. Rapa was used as an autophagy agonist. Data were displayed as *mean ± SD*. *Unpaired Student's t tests* were employed for two-variable comparisons. **, p< 0.05; **, p< 0.01; ***, p< 0.001; ****, p< 0.0001*; ns, no significance in comparison with control group.

**
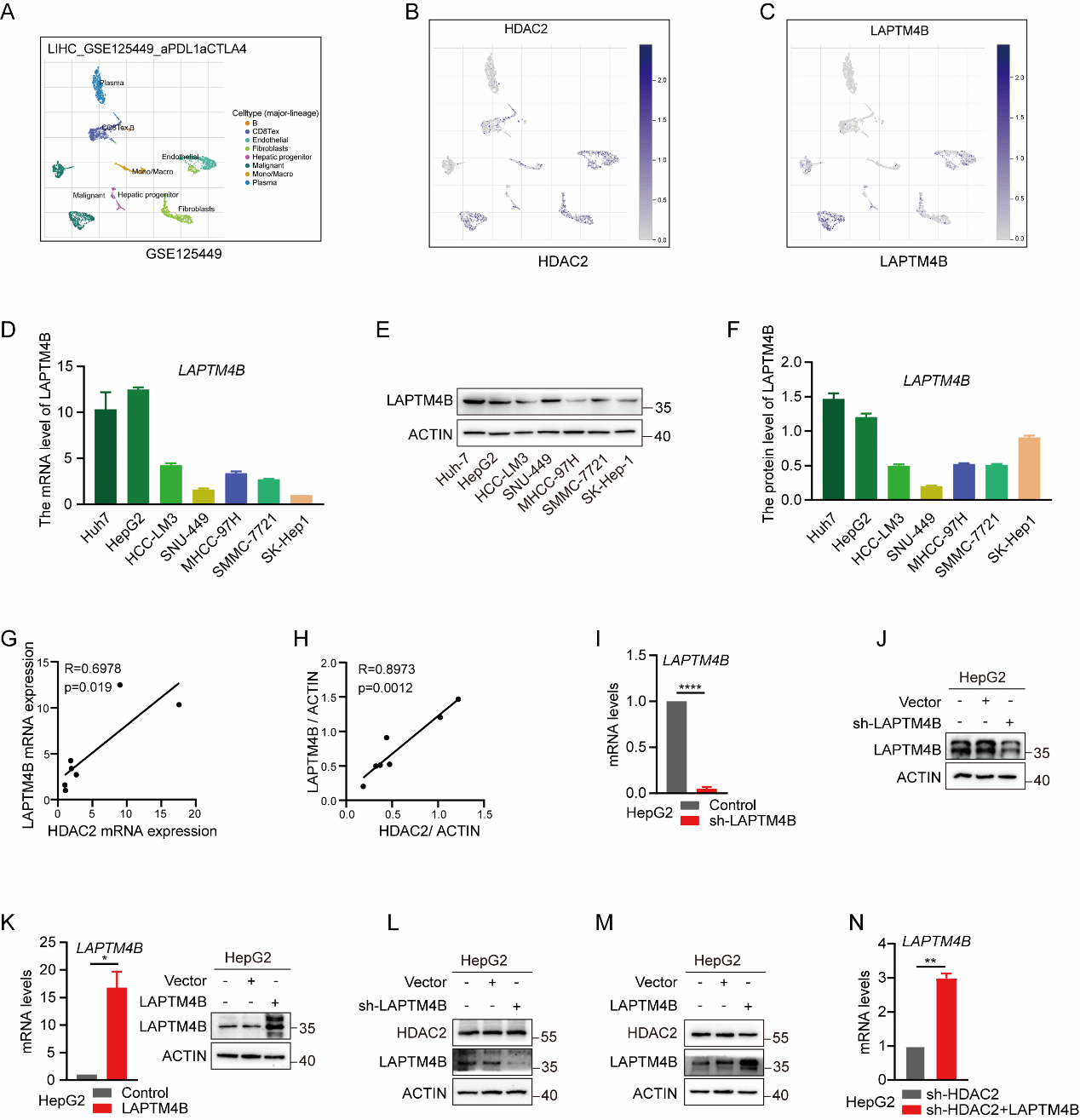
**

**Figure S3. HDAC2 regulates LAPTM4B to promote both autophagy and its associated malignancy in HCC, Related to Figure 4 and Figure 5**

(**A**) (**B**) (**C**) HDAC2 and LAPTM4B shared similar expression profiles in HCC cells from the single-cell sequencing data of HCC in GEO database (GSE125449). (**D**) (**E**) (**F**) The mRNA and protein level of LAPTM4B in seven HCC cell lines (Huh7, HepG2, HCC-LM3, SNU-449, MHCC-97H, SMMC-7721, and SK-Hep1) were detected by qRT-PCR and western blotting. (**G**) (**H**) Correlation between the homeodomain expression of HDAC2 and LAPTM4B in seven human HCC cell lines. (**I**) (**J**) (**K**) Detection of the knockdown or overexpression efficiency of LAPTM4B in HepG2 cells by qRT-PCR and western blotting. (**L**) (**M**) Western blotting assay of HDAC2 expression in the LAPTM4B knockdown or overexpressing HCC cells. (**N**) Detection of the overexpression efficiency of LAPTM4B in HDAC2-knockdown HepG2 cells by qRT-PCR. All data were subjected to at least three separate experiments. Data were displayed as *mean ± SD*. *Unpaired Student's t tests* were employed for two-variable comparisons. **, p< 0.05; **, p< 0.01; ***, p< 0.001; ****, p< 0.0001*; ns, no significance in comparison with control group.


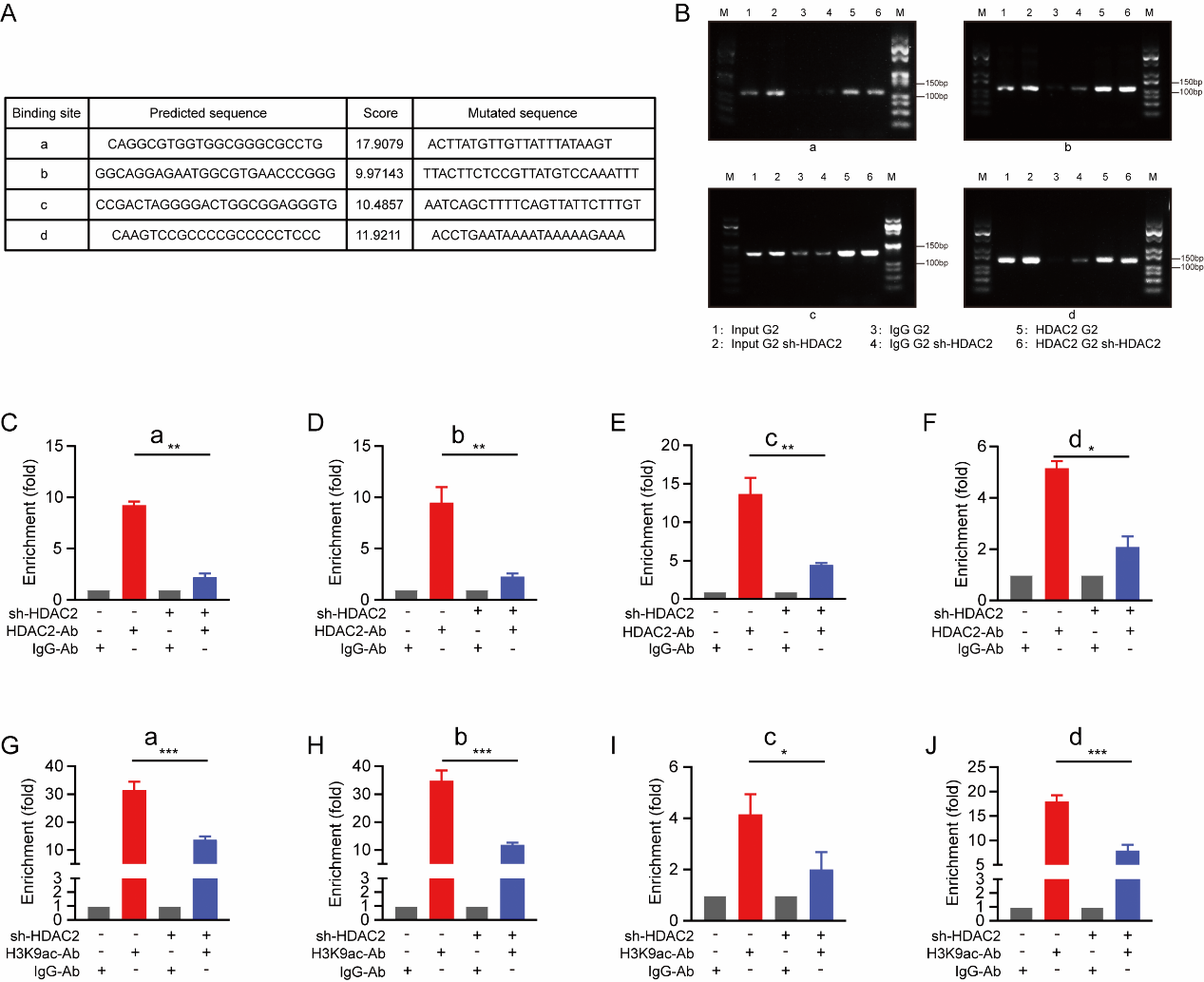


**Figure S4. HDAC2 enhances autophagy in HCC cells by transcriptionally activating LAPTM4B, Related to Figure 5**

(**A**) Sequences of putative HDAC2 binding sites (HBS) in the LAPTM4B promoter predicted from hTFtarget database and corresponding mutant sequences. (**B**) (**C**) (**D**) (**E**) (**F**) ChIP assays in HDAC2 knockdown HepG2 cells to evaluate the binding of HDAC2 to the LAPTM4B promoter. (**G**) (**H**) (**I**) (**J**) ChIP assay for H3K9ac was performed in HDAC2 knockdown HepG2 cells to examine the histone acetylation levels of the four binding sites of HDAC2 and LAPTM4B. All data were subjected to at least three separate experiments. Data were displayed as *mean ± SD*. *Unpaired Student's t tests* were employed for two-variable comparisons. **, p< 0.05; **, p< 0.01; ***, p< 0.001; ****, p< 0.0001;* ns, no significance in comparison with control group.


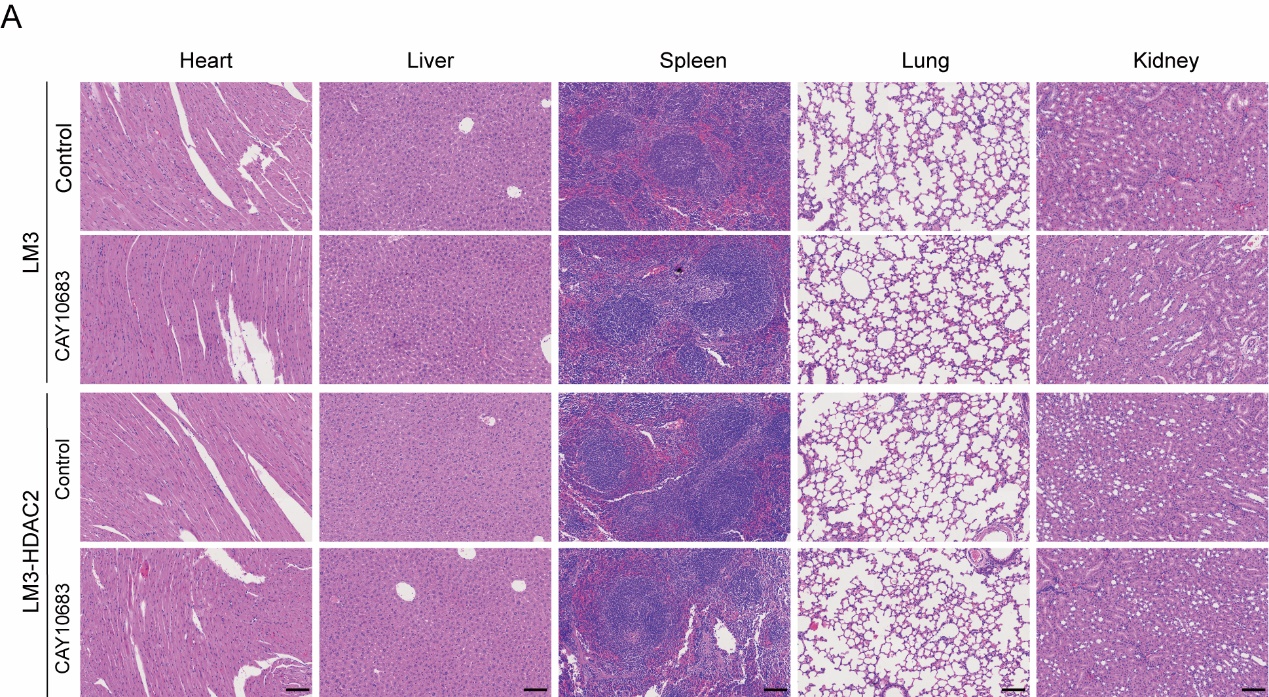


**Figure S5. Effects of CAY10683 on major organs in tumor-bearing mice, Related to Figure 7**

(**A**) HE staining of major organs (Heart, Liver, Spleen, Lung, Kidney) in tumor-bearing mice treated or not with CAY10683 intratumoral injection. Scale bar = 100µm.

**Supplementary Tables and Legends**

**Table S1. HDAC2 is positively correlated with LAPTM4B in HCC, Related to Figure 8**

|  |  | LAPTM4B expression | | Total | p value |
| --- | --- | --- | --- | --- | --- |
|  |  | High | Low |  | (Chi-square test) |
| HDAC2 expression | High | 45 | 6 | 51 | < 0.001 *** |
|  | Low | 5 | 49 | 54 |  |
| Total |  | 50 | 55 | 105 |  |

Statistically significant; ****, p< 0.001*.

**Table S2. Clinical relevance of HDAC2 in HCC, Related to Figure 8**

| Groups | HDAC2 expression | | n | Positive  (%) | p value  (Chi-square test) |  |
| --- | --- | --- | --- | --- | --- | --- |
|  |  |  |  |  |  |  |
|  | Low | High |  |  |  |  |
| Sex |  |  |  |  |  |  |
| Male | 46 | 45 | 91 | 86.67 | 0.646 |  |
| Female | 8 | 6 | 14 | 13.33 |  |  |
| Age (years) |  |  |  |  |  |  |
| ≤60 | 29 | 37 | 66 | 62.86 | 0.046 |  |
| >60 | 25 | 14 | 39 | 37.14 |  |  |
| Tumor size (cm) |  |  |  |  |  |  |
| <10 | 34 | 31 | 65 | 61.90 | 0.818 |  |
| ≥10 | 20 | 20 | 40 | 38.10 |  |  |
| HBsAg |  |  |  |  |  |  |
| No | 18 | 16 | 34 | 32.38 | 0.830 |  |
| Yes | 36 | 35 | 71 | 67.62 |  |  |
| Cirrhosis |  |  |  |  |  |  |
| No | 21 | 19 | 40 | 38.10 | 0.863 |  |
| Yes | 33 | 32 | 65 | 61.90 |  |  |
| Microvascular invasion |  |  |  |  |  |  |
| No | 16 | 4 | 20 | 19.05 | 0.004 ** |  |
| Yes | 38 | 47 | 85 | 80.95 |  |  |
| Edmondson classification |  |  |  |  |  |  |
| Ⅰ+Ⅱ | 24 | 10 | 34 | 32.38 | 0.007 ** |  |
| Ⅲ+Ⅳ | 30 | 41 | 71 | 67.62 |  |  |
| Microsatellites |  |  |  |  |  |  |
| No | 31 | 27 | 58 | 55.24 | 0.646 |  |
| Yes | 23 | 24 | 47 | 44.76 |  |  |
| Total | 54 | 51 | 105 | 100 |  |  |

Statistically significant; ***, p< 0.01*.

**Table S3. qPCR primers**

| **Primer** |  | **Sequence (5'→3')** |
| --- | --- | --- |
| h-18S | Forward | AGAAACGGCTACCACATCCA |
|  | Reverse | CACCAGACTTGCCCTCCA |
| h-HDAC2 | Forward | CGTACAGTCAAGGAGGCGG |
|  | Reverse | CTTCGGCAGTGGCTTTATGGG |
| h-LAPTM4B | Forward | GACGCGGTTCTACTCCAACAGC |
|  | Reverse | TCAGATACCAGACGCCGAGCAG |
| h-ATG3 | Forward | GCCGTTAAAGAGATCACACTGGAA |
|  | Reverse | TTCACCGCCAGCATCAGTTT |
| h-ATG5 | Forward | GCTTCGAGATGTGTGGTTTGGA |
|  | Reverse | TGGATAATGCCATTTCAGTGGTG |
| h-ATG7 | Forward | CTGTTCACCCAAAGTTCTTG |
|  | Reverse | TCTAAGAAGGAATGTGAGGAG |
| h-Beclin-1 | Forward | GAGGGATGGAAGGGTCTA |
|  | Reverse | GCCTGGGCTGTGGTAAGT |
| h-LC3 | Forward | GCCGCACCTTCGAACAAAGA |
|  | Reverse | TGGTGTGGAGACGCTGACC |
| h-LAPTM4B 2 (ChIP) | Forward | TAGAAGGGAAGAAAATCG |
|  | Reverse | GTAAATCCATCAGCGTGC |
| h-LAPTM4B 3 (ChIP) | Forward | TAGGGGACTGGCGGAGGGTG |
|  | Reverse | ATCTGGGGAGGGGGCGGG |
| h-LAPTM4B 7 (ChIP) | Forward | CCCCGTCTCTACTGAAAATAC |
|  | Reverse | GGTTCACGCCATTCTCCT |
| h-LAPTM4B 8 (ChIP) | Forward | GAGGCTGAGGCAGGAGAA |
|  | Reverse | GAGTCTTGCTCTGTCACCC |

**Table S4. Primary antibodies for western blotting, immunohistochemistry, immunofluorescence and ChIP.**

| **Antibodies** | **Source** | **Application and dilution ratio** |
| --- | --- | --- |
| Anti-HDAC2 antibody [Y461] | ab32117; abcam | 1:2000 for WB; 1:500 for IHC; 1:300 for IF |
| HDAC2 (D6S5P) Rabbit mAb | #57156; CST | 1:50 for ChIP |
| Anti-LAPTM4B antibody [CL5289] | ab242376; abcam | 1:1000 for WB; 1:500 for IHC; 1:200 for IF |
| Atg3 Antibody | #3415; CST | 1:1000 for WB |
| ATG3 Rabbit pAb | bs-4013R; Bioss | 1:100 for IHC |
| Atg5 (D5F5U) Rabbit mAb | #12994; CST | 1:1000 for WB |
| Atg7 (D12B11) Rabbit mAb | #8558; CST | 1:1000 for WB |
| Beclin 1 Antibody | T55092; Abmart | 1:1000 for WB; 1:100 for IHC |
| LC3A/B Antibody | AF5402; Affinity | 1:1000 for WB; 1:100 for IF |
| P62, SQSTM1 Polyclonal antibody | 18420-1-AP; proteintech | 1:5000 for WB; 1:1500 for IF |
| Rabbit IgG control Polyclonal antibody | 30000-0-AP; proteintech | 2 µg per ChIP |
| Histone H3K9ac antibody (mAb) | #61252; Active Motif | 2 µg per ChIP |
| Histone H3K27ac antibody (pAb) | #39134; Active Motif | 2 µg per ChIP |
| Ki67 | RMA-0731; MXB | Ready-to-use antibodies for IHC |
| β-Actin (13E5) Rabbit mAb | #4970; CST | 1:1000 for WB |
| HRP Conjugated AffiniPure Goat AntiRabbit IgG (H+L) | BA1054; Boster | 1:5000 for WB |
| Goat anti-Mouse IgG (H+L) Secondary Antibody; HRP | #31430; Invitrogen | 1:5000 for WB |
| Goat Anti-Rabbit IgG H&L (Alexa Fluor® 594) | ab150080; abcam | 1:1000 for IHC |
| Goat Anti-Rabbit IgG H&L (Alexa Fluor® 488) | ab150077; abcam | 1:1000 for IHC |
