# Supplementary material for: HDAC2 promotes autophagy-associated HCC malignant progression by transcriptionally activating LAPTM4B
Source: Cell Death Dis. 2024 Aug 15;15(8):593. doi: 10.1038/s41419-024-06981-3 (PMC11327261; doi:10.1038/s41419-024-06981-3)
Supplement: Supplementary file 2 — Original western blots for HDAC2 promotes autophagy-associated HCC malignant progression by transcriptionally activating LAPTM4B [file 41419_2024_6981_MOESM2_ESM.pptx]

## Slide 1
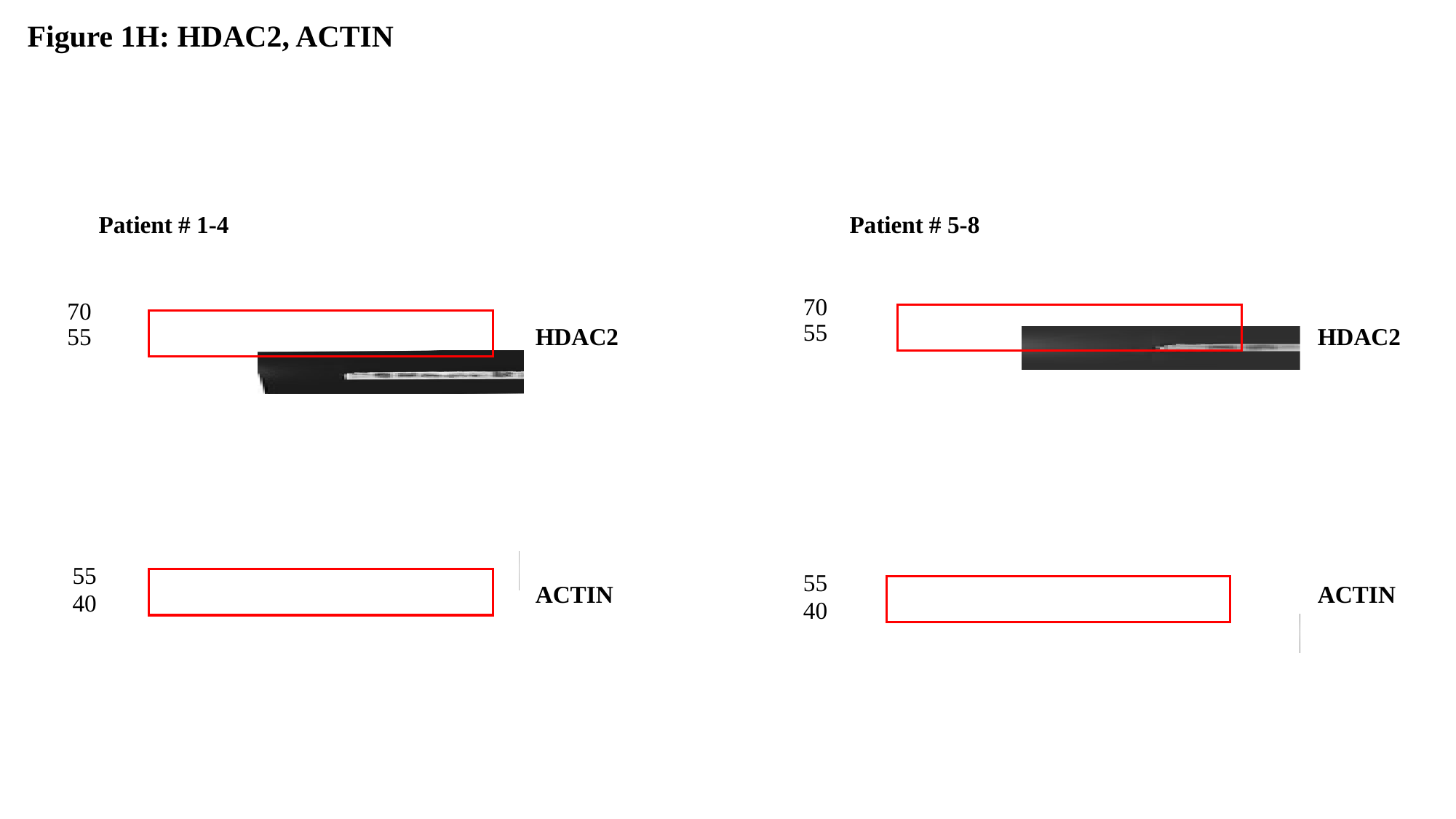

Figure 1H: HDAC2, ACTIN
Patient # 1-4
Patient # 5-8
70
70
55
55
HDAC2
HDAC2
55
55
ACTIN
ACTIN
40
40

## Slide 2
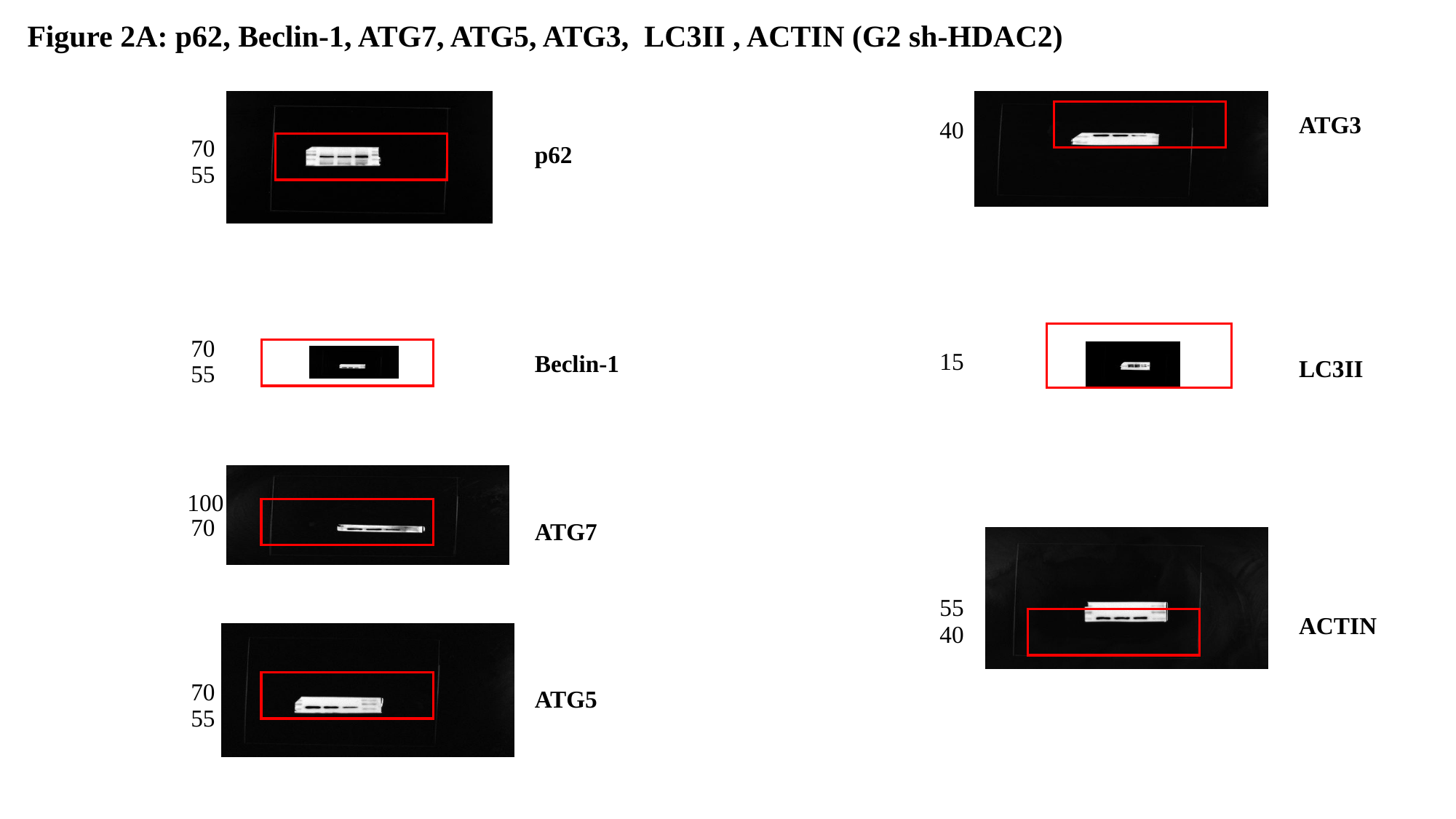

Figure 2A: p62, Beclin-1, ATG7, ATG5, ATG3, LC3II , ACTIN (G2 sh-HDAC2)
ATG3
40
70
p62
55
70
15
Beclin-1
LC3II
55
100
70
ATG7
55
ACTIN
40
70
ATG5
55

## Slide 3
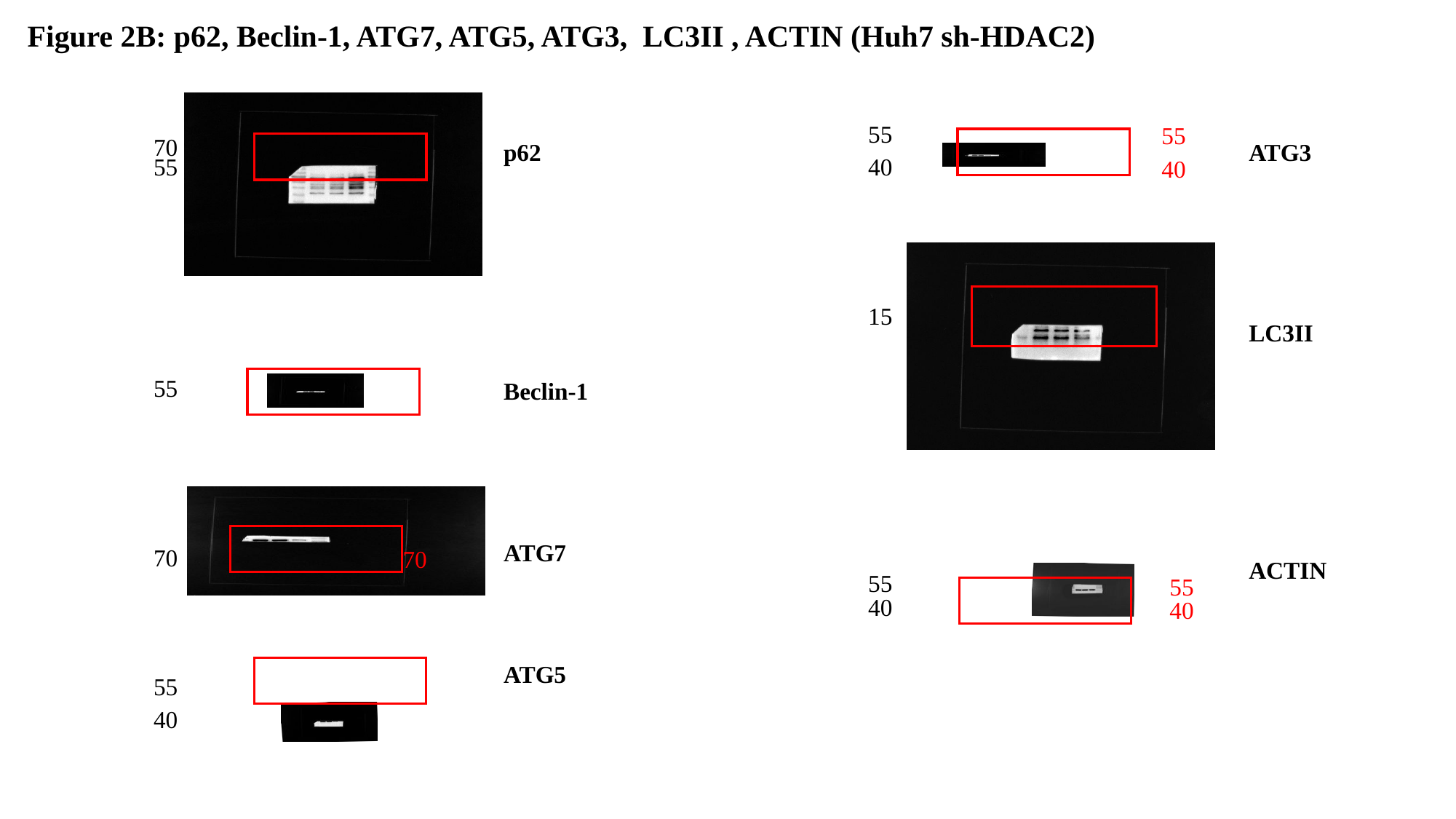

Figure 2B: p62, Beclin-1, ATG7, ATG5, ATG3, LC3II , ACTIN (Huh7 sh-HDAC2)
55
55
70
ATG3
p62
55
40
40
15
LC3II
55
Beclin-1
ATG7
70
70
ACTIN
55
55
40
40
ATG5
55
40

## Slide 4
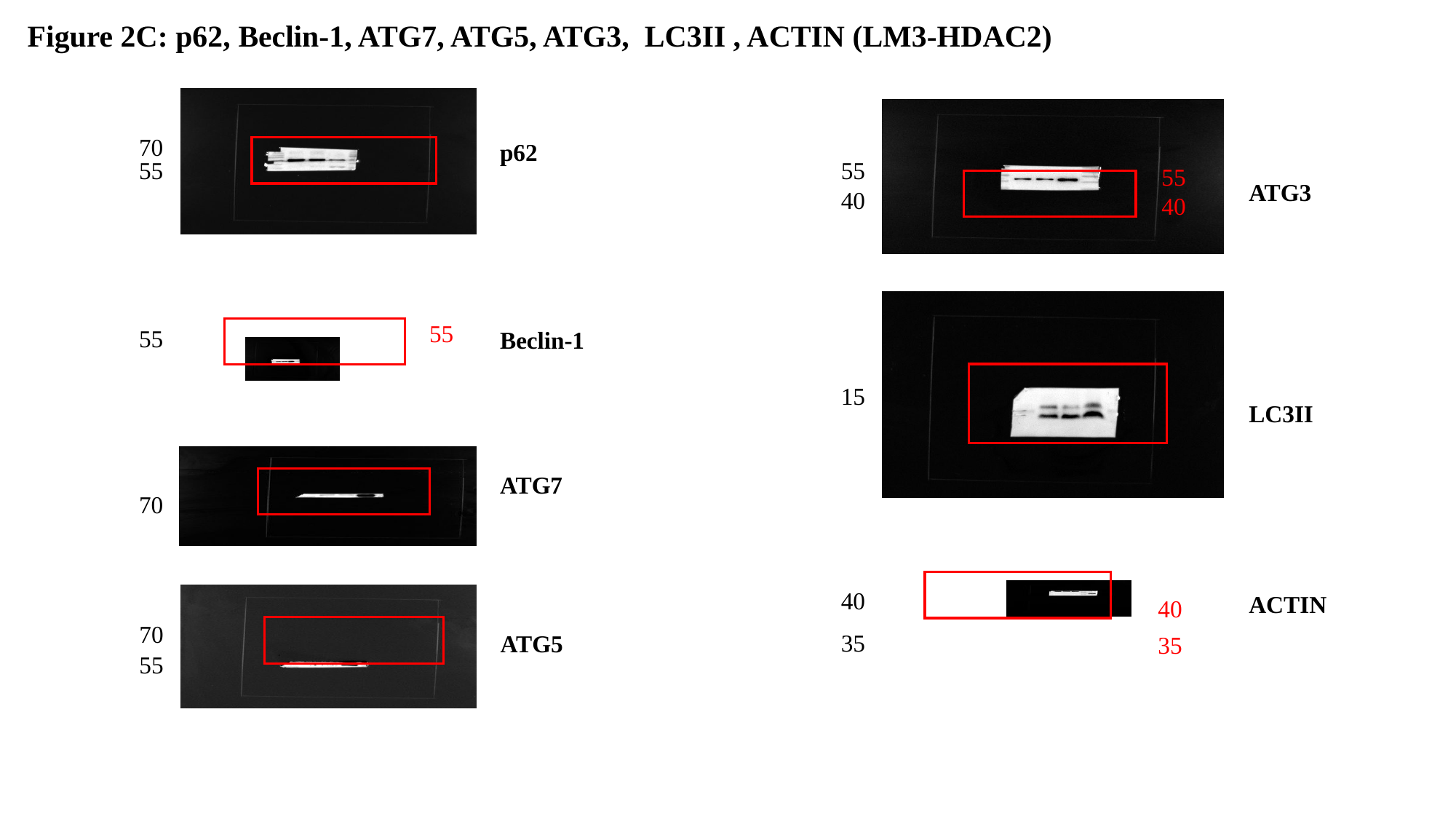

Figure 2C: p62, Beclin-1, ATG7, ATG5, ATG3, LC3II , ACTIN (LM3-HDAC2)
70
p62
55
55
55
ATG3
40
40
55
55
Beclin-1
15
LC3II
ATG7
70
40
ACTIN
40
70
35
ATG5
35
55

## Slide 5
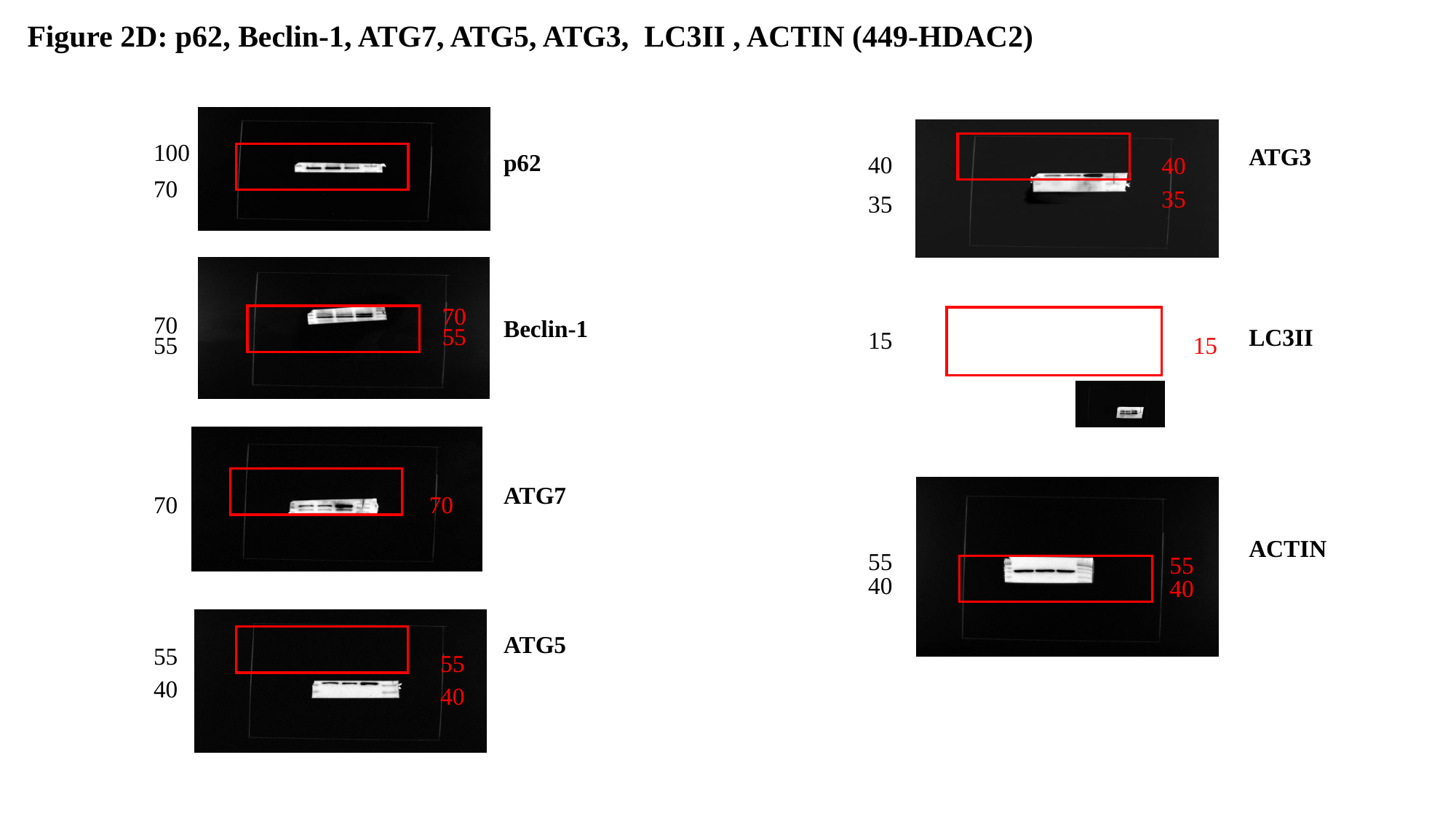

Figure 2D: p62, Beclin-1, ATG7, ATG5, ATG3, LC3II , ACTIN (449-HDAC2)
100
ATG3
p62
40
40
70
35
35
70
70
Beclin-1
55
LC3II
15
15
55
ATG7
70
70
ACTIN
55
55
40
40
ATG5
55
55
40
40

## Slide 6
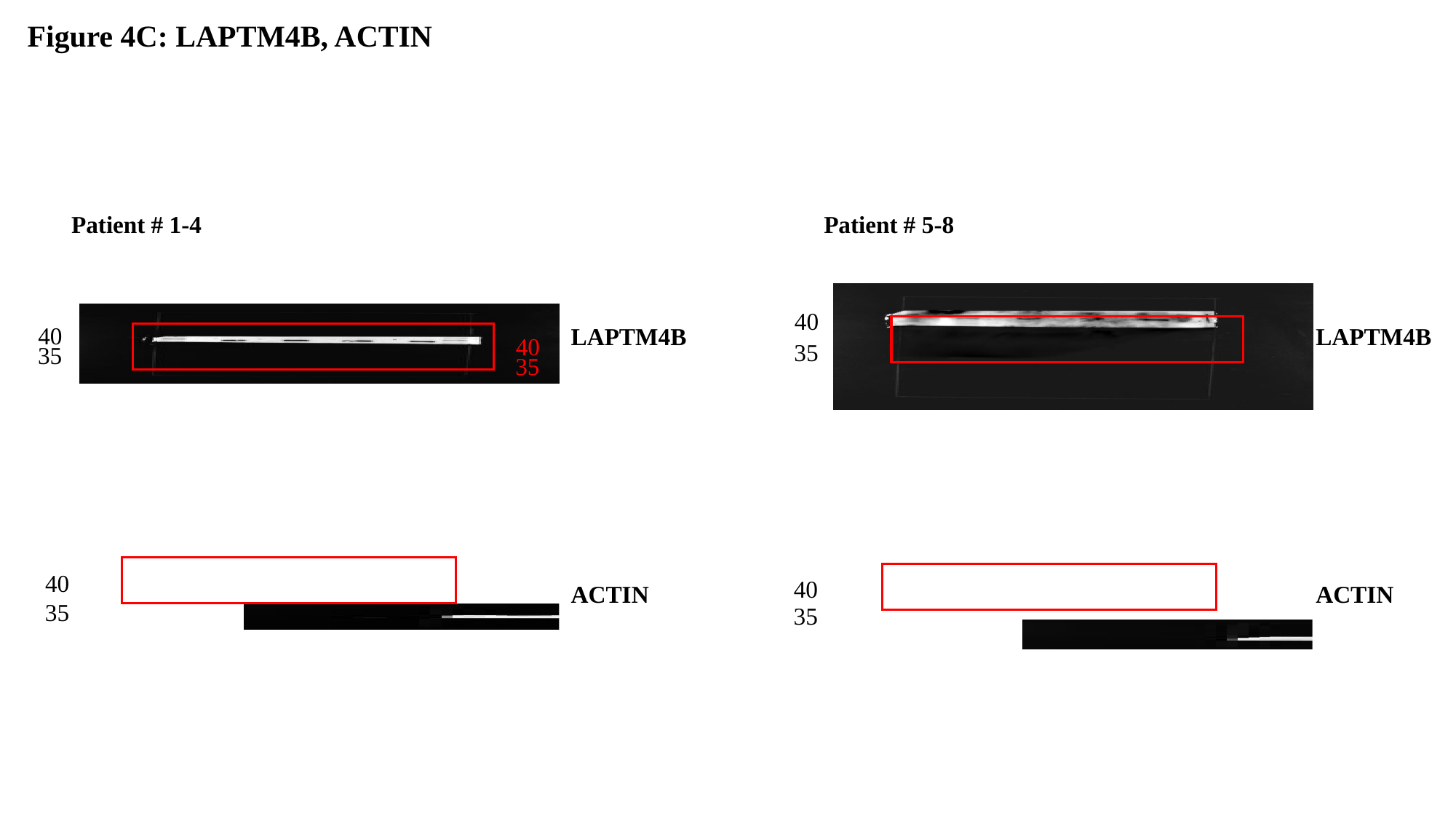

Figure 4C: LAPTM4B, ACTIN
Patient # 1-4
Patient # 5-8
40
40
LAPTM4B
LAPTM4B
40
35
35
35
40
40
ACTIN
ACTIN
35
35

## Slide 7
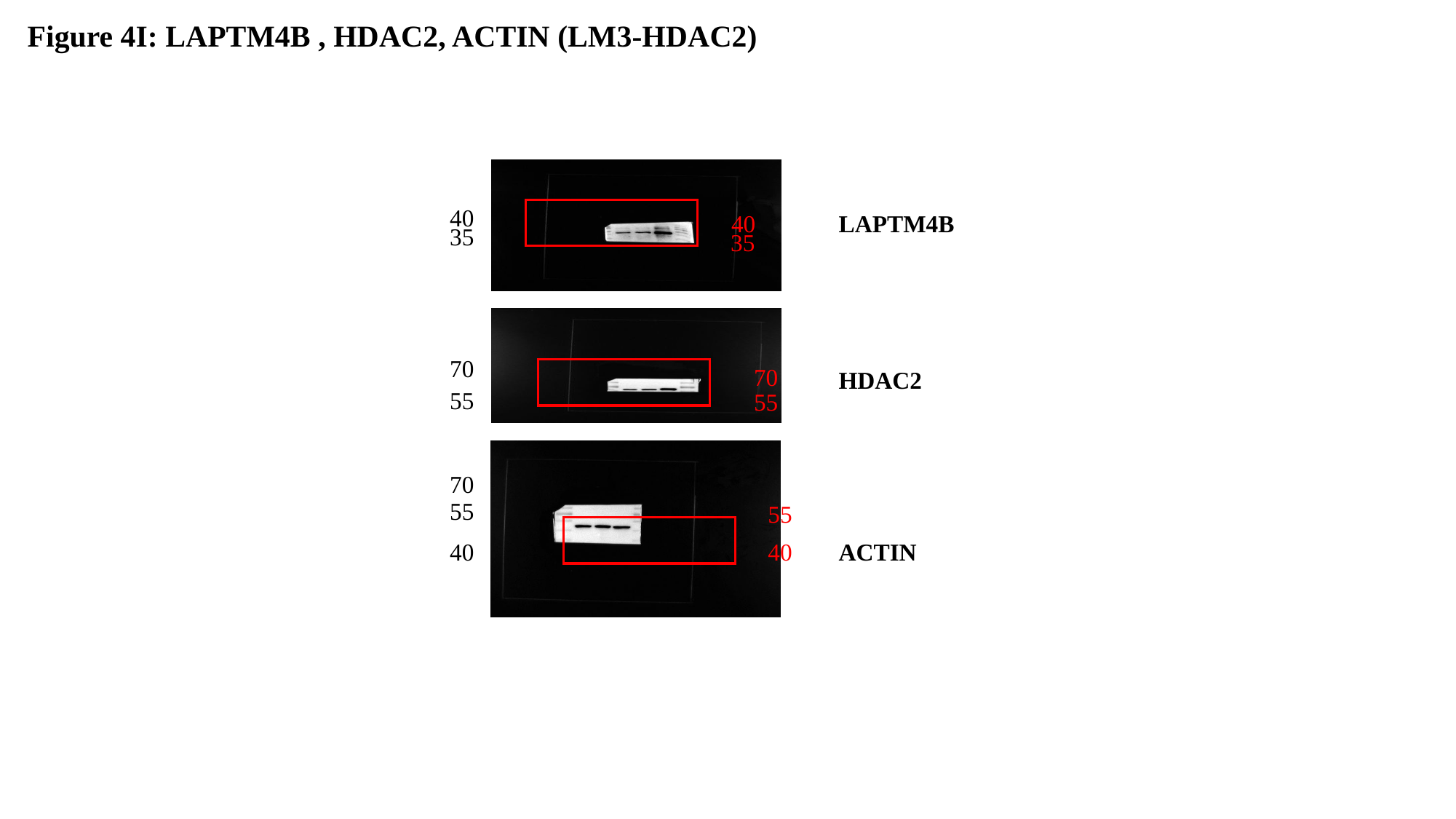

Figure 4I: LAPTM4B , HDAC2, ACTIN (LM3-HDAC2)
40
40
LAPTM4B
35
35
70
70
HDAC2
55
55
70
55
55
40
ACTIN
40

## Slide 8
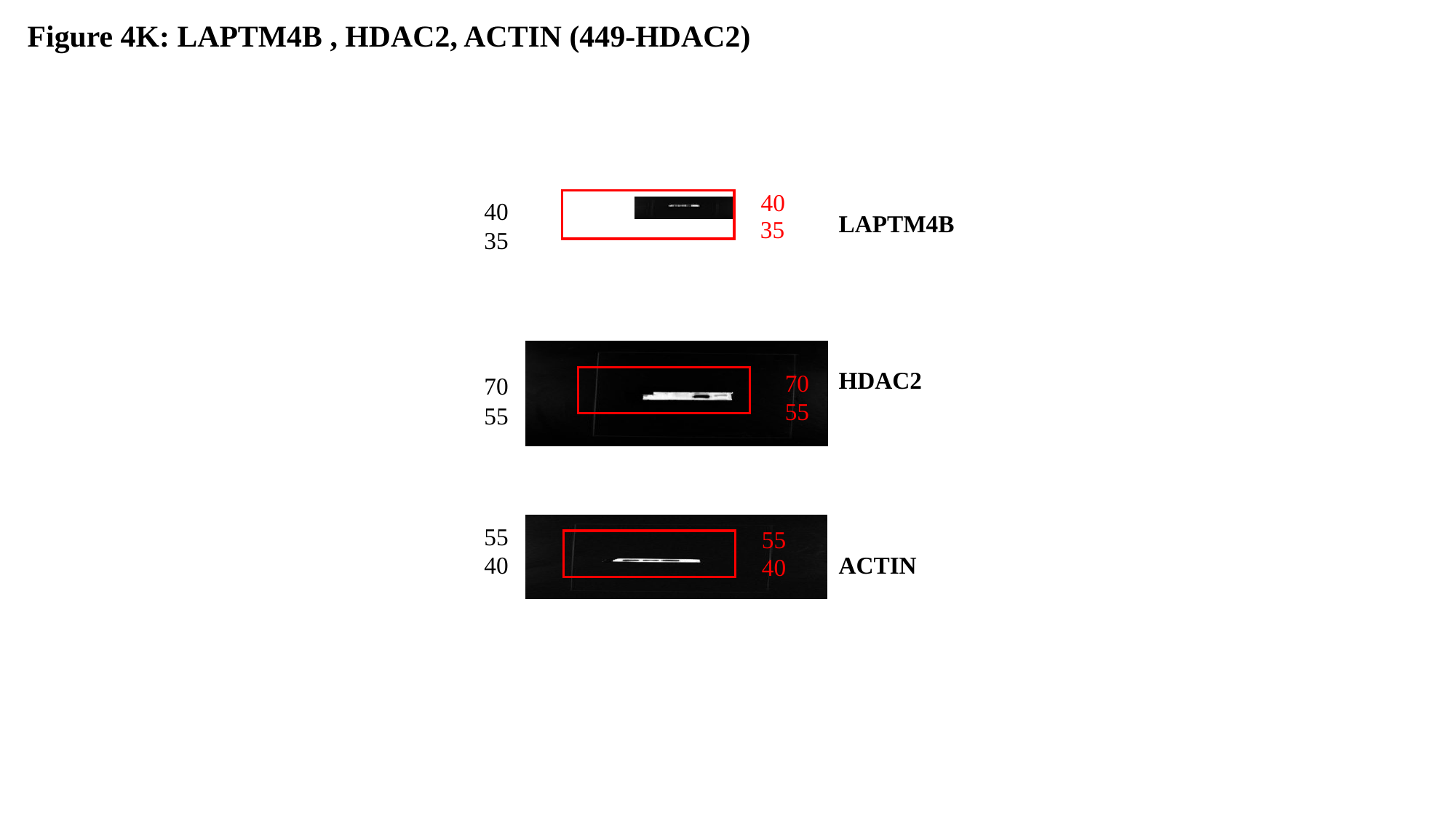

Figure 4K: LAPTM4B , HDAC2, ACTIN (449-HDAC2)
40
40
LAPTM4B
35
35
HDAC2
70
70
55
55
55
55
40
ACTIN
40

## Slide 9
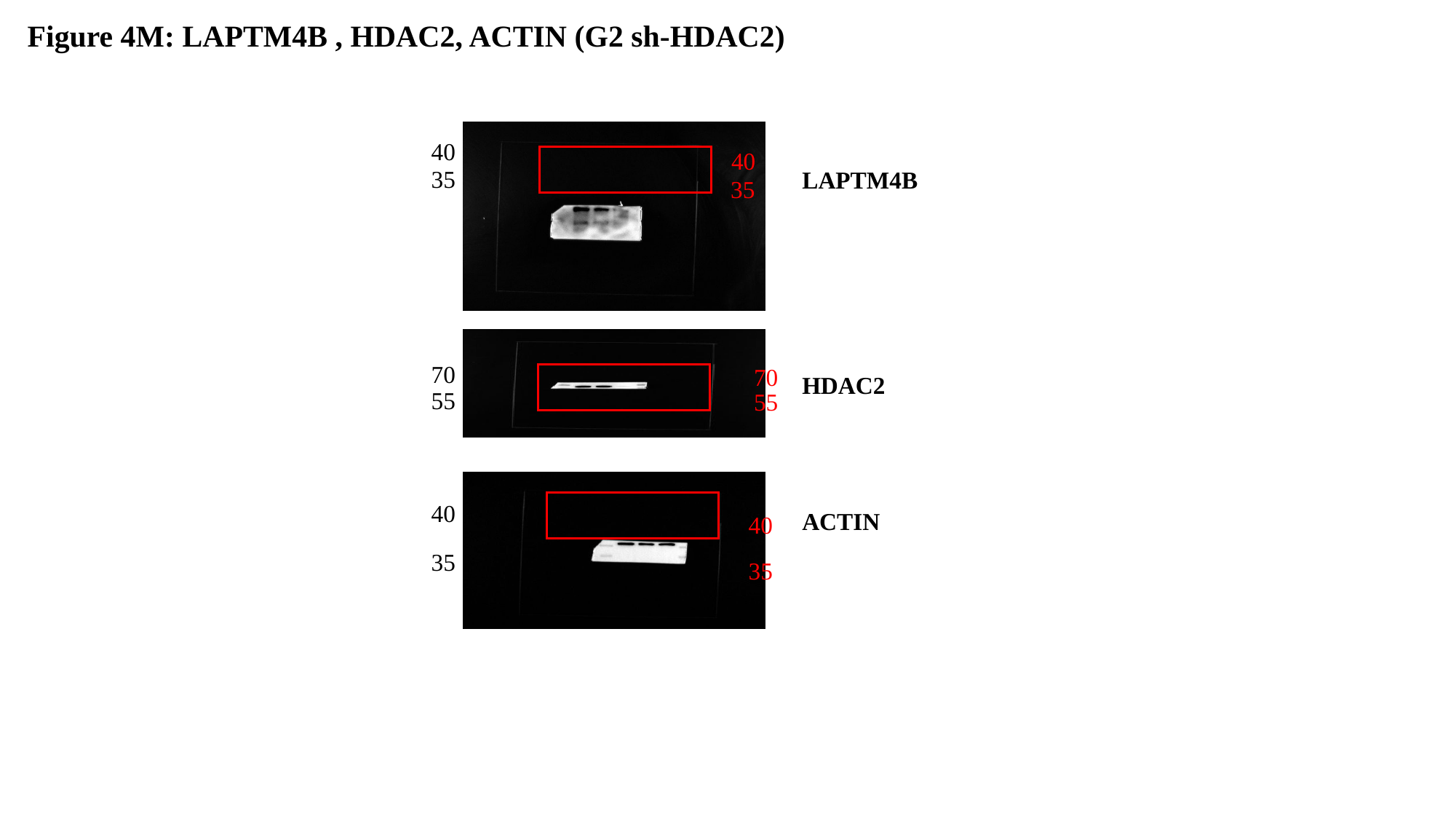

Figure 4M: LAPTM4B , HDAC2, ACTIN (G2 sh-HDAC2)
40
40
35
LAPTM4B
35
70
70
HDAC2
55
55
40
ACTIN
40
35
35

## Slide 10
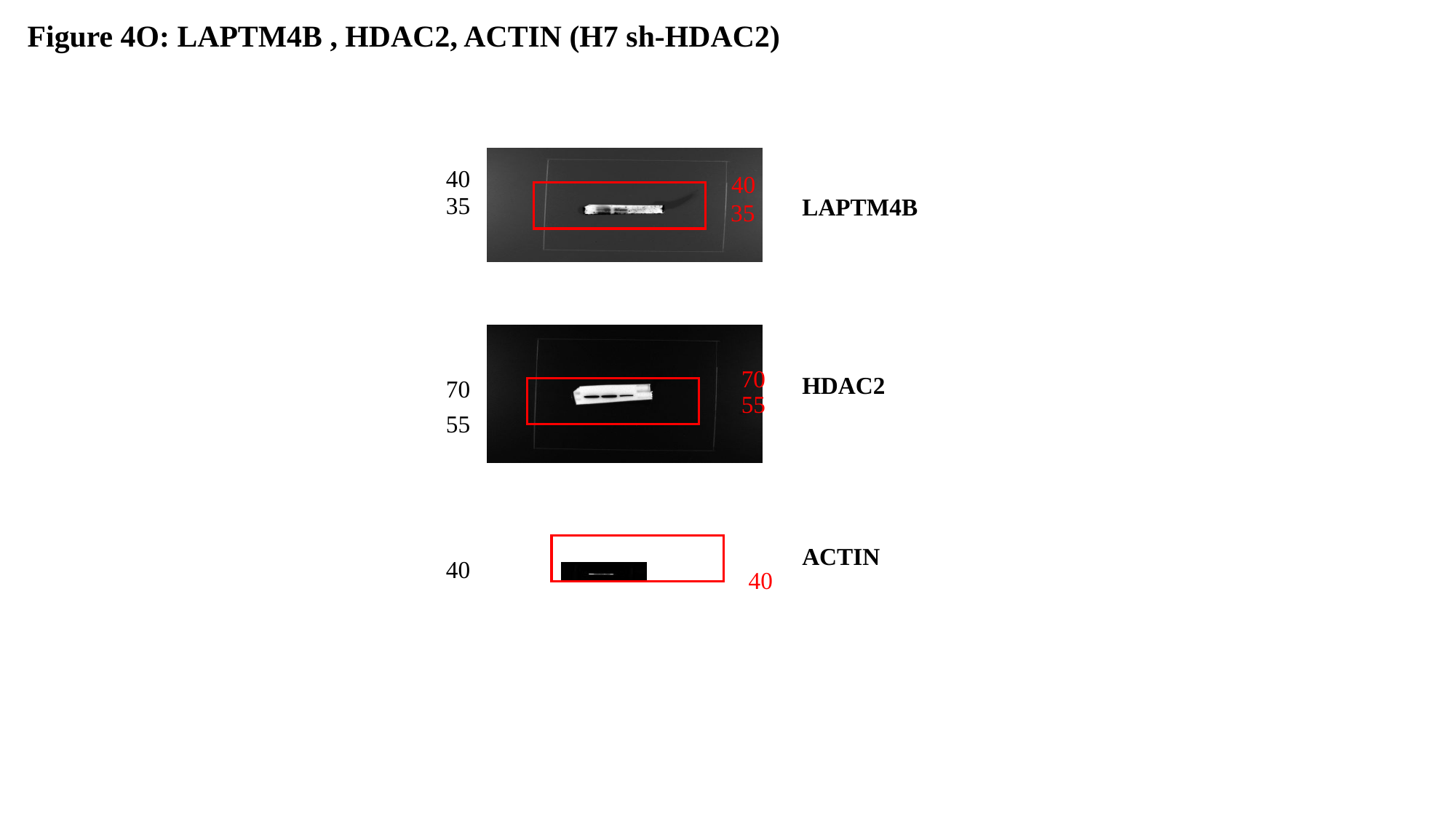

Figure 4O: LAPTM4B , HDAC2, ACTIN (H7 sh-HDAC2)
40
40
35
LAPTM4B
35
70
HDAC2
70
55
55
ACTIN
40
40

## Slide 11
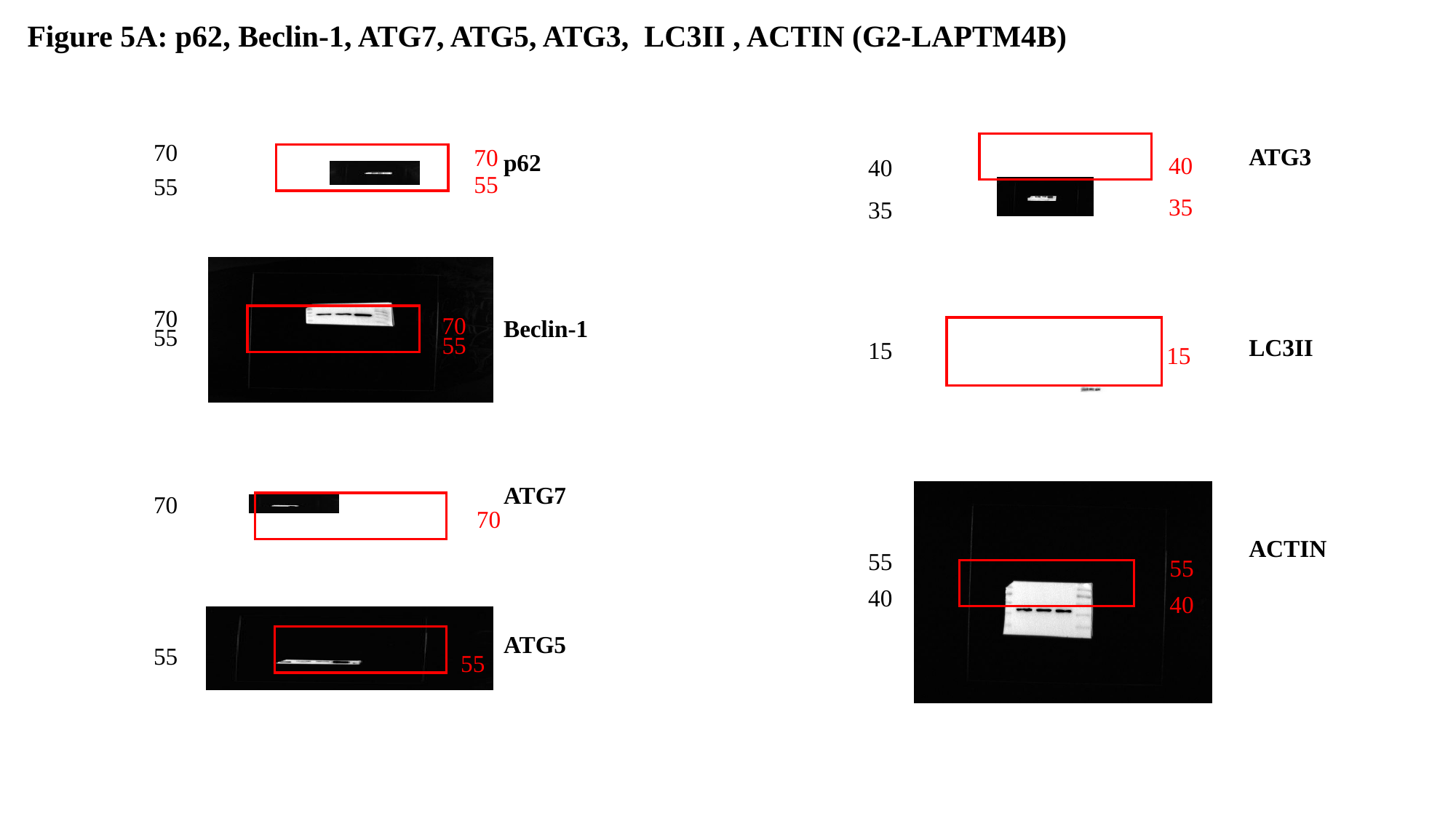

Figure 5A: p62, Beclin-1, ATG7, ATG5, ATG3, LC3II , ACTIN (G2-LAPTM4B)
70
ATG3
70
p62
40
40
55
55
35
35
70
70
Beclin-1
55
55
LC3II
15
15
ATG7
70
70
ACTIN
55
55
40
40
ATG5
55
55

## Slide 12
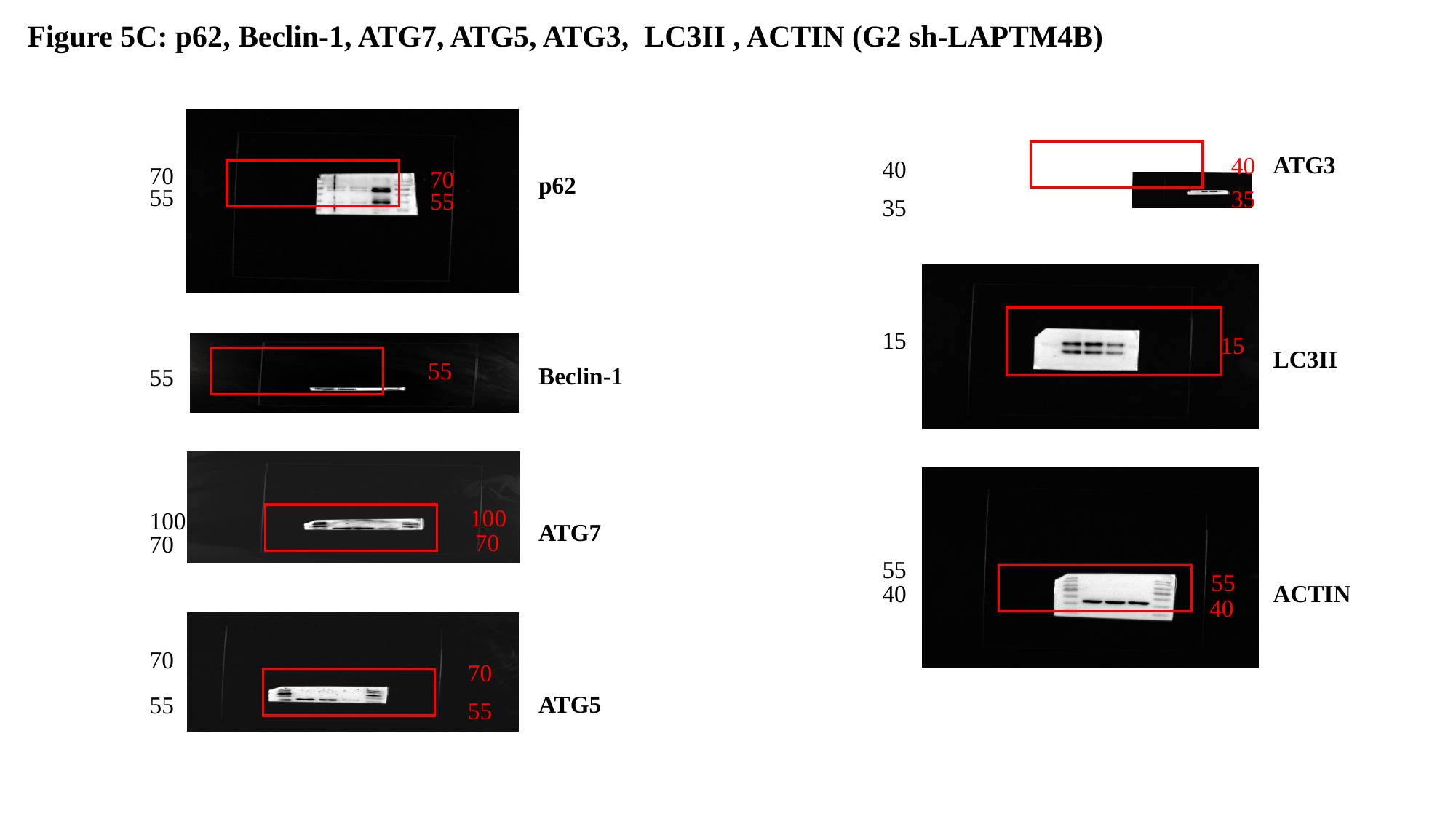

Figure 5C: p62, Beclin-1, ATG7, ATG5, ATG3, LC3II , ACTIN (G2 sh-LAPTM4B)
ATG3
40
40
70
70
p62
55
35
55
35
15
15
LC3II
55
Beclin-1
55
100
100
ATG7
70
70
55
55
40
ACTIN
40
70
70
ATG5
55
55

## Slide 13
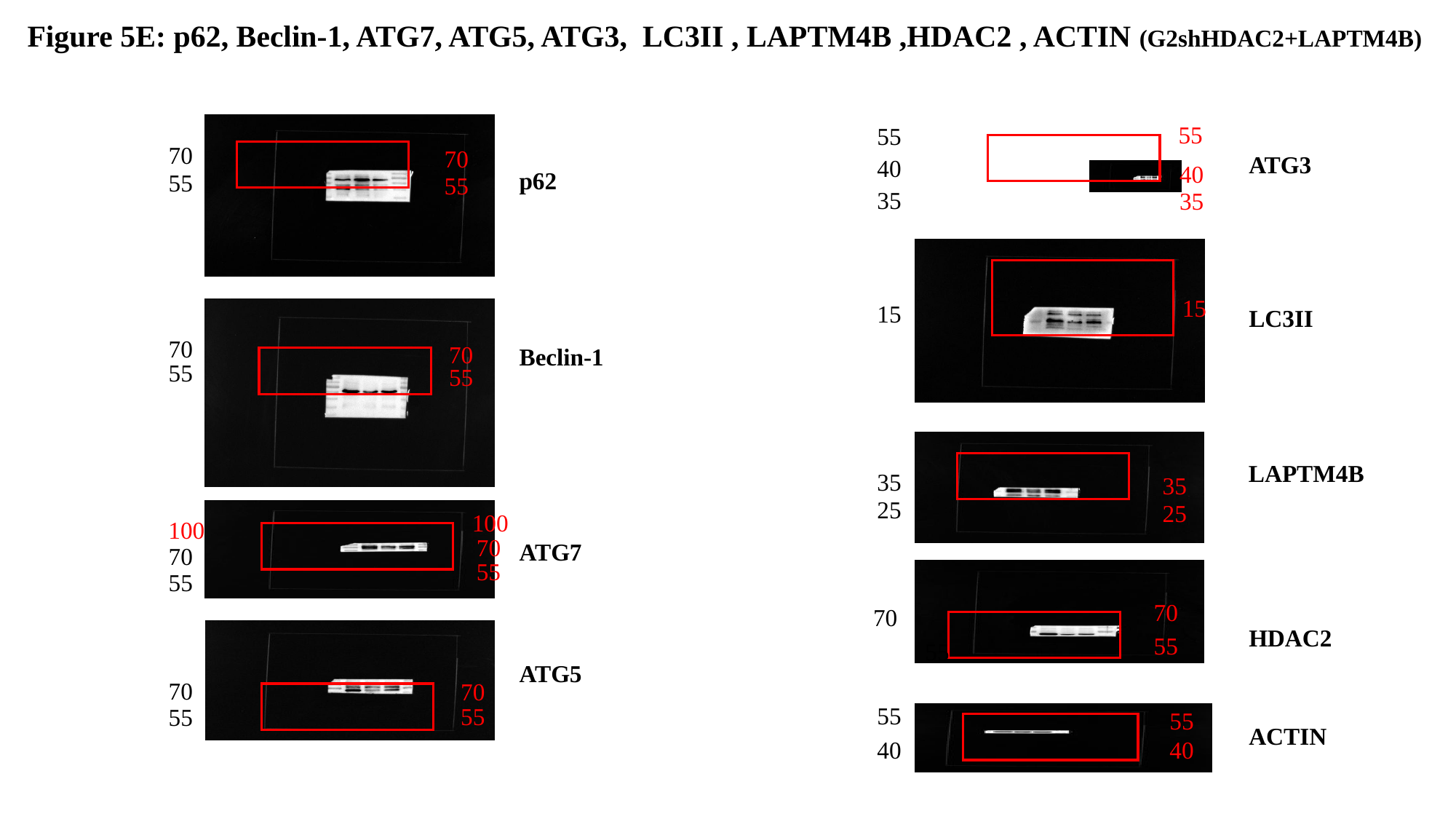

Figure 5E: p62, Beclin-1, ATG7, ATG5, ATG3, LC3II , LAPTM4B ,HDAC2 , ACTIN (G2shHDAC2+LAPTM4B)
55
55
70
70
ATG3
40
40
p62
55
55
35
35
15
15
LC3II
70
70
Beclin-1
55
55
LAPTM4B
35
35
25
25
100
100
70
ATG7
70
55
55
70
70
HDAC2
55
55
ATG5
70
70
55
55
55
55
ACTIN
40
40

## Slide 14
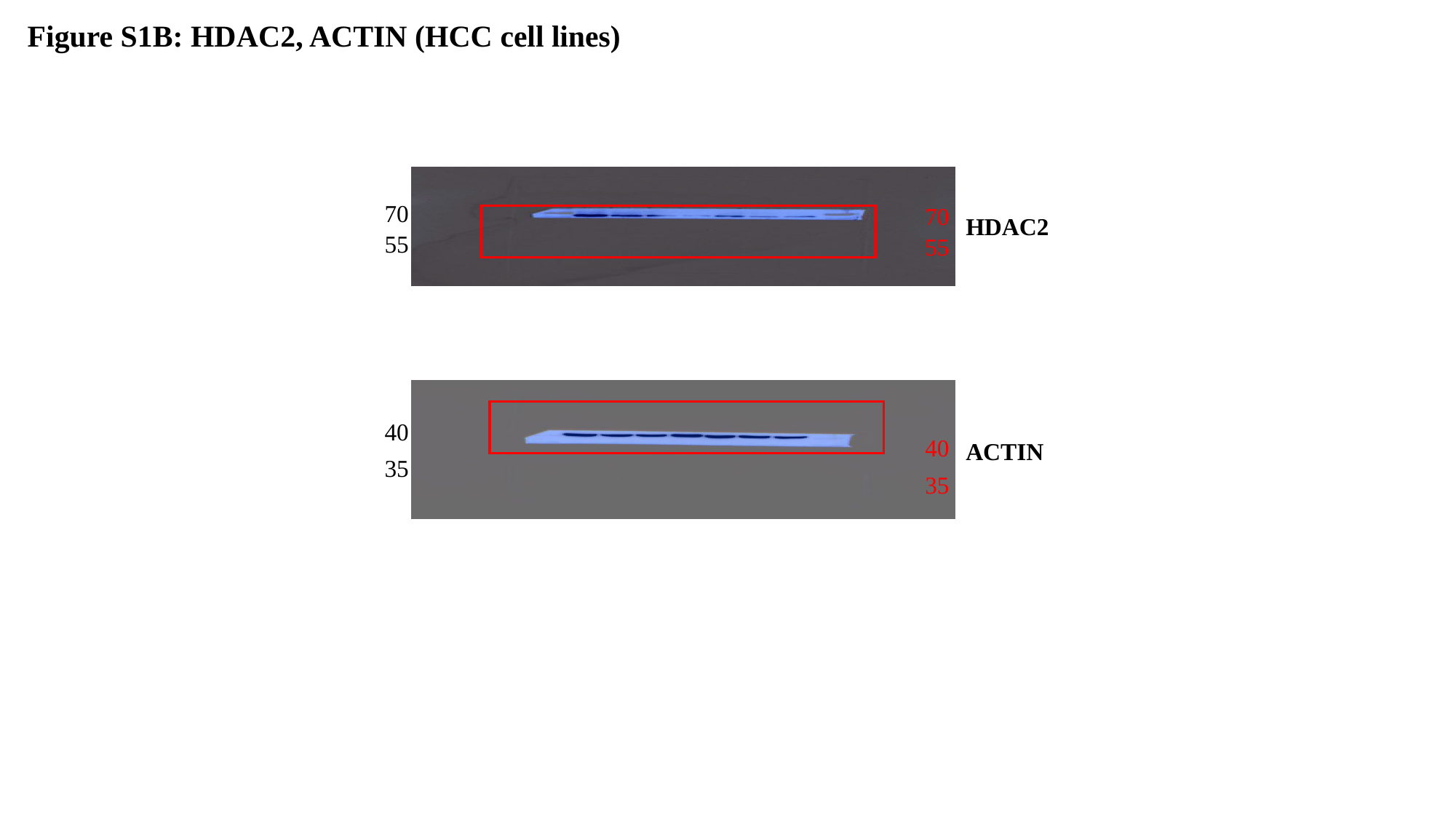

Figure S1B: HDAC2, ACTIN (HCC cell lines)
70
70
HDAC2
55
55
40
40
ACTIN
35
35

## Slide 15
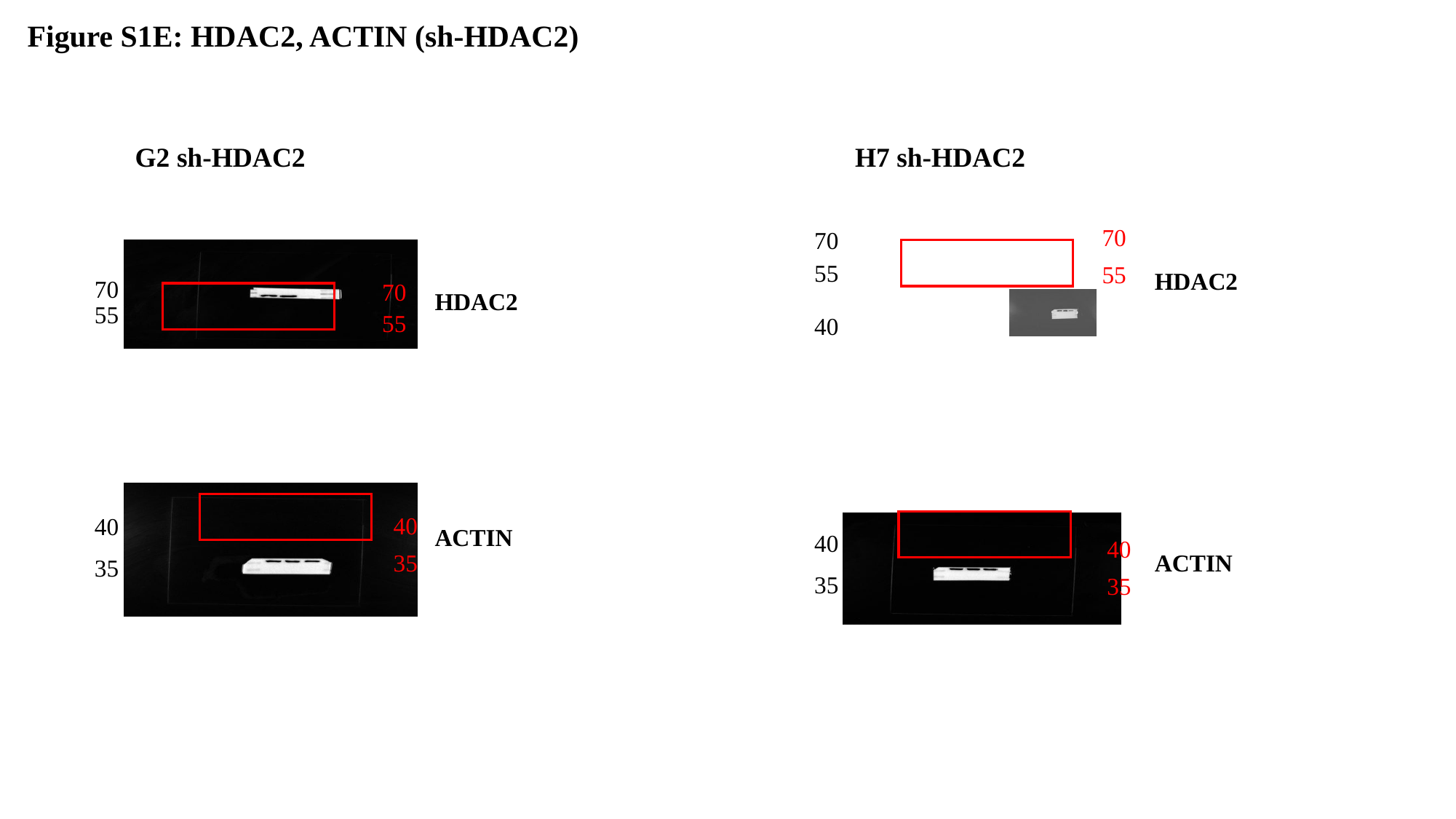

Figure S1E: HDAC2, ACTIN (sh-HDAC2)
G2 sh-HDAC2
H7 sh-HDAC2
70
70
55
55
HDAC2
70
70
HDAC2
55
55
40
40
40
ACTIN
40
40
35
ACTIN
35
35
35

## Slide 16
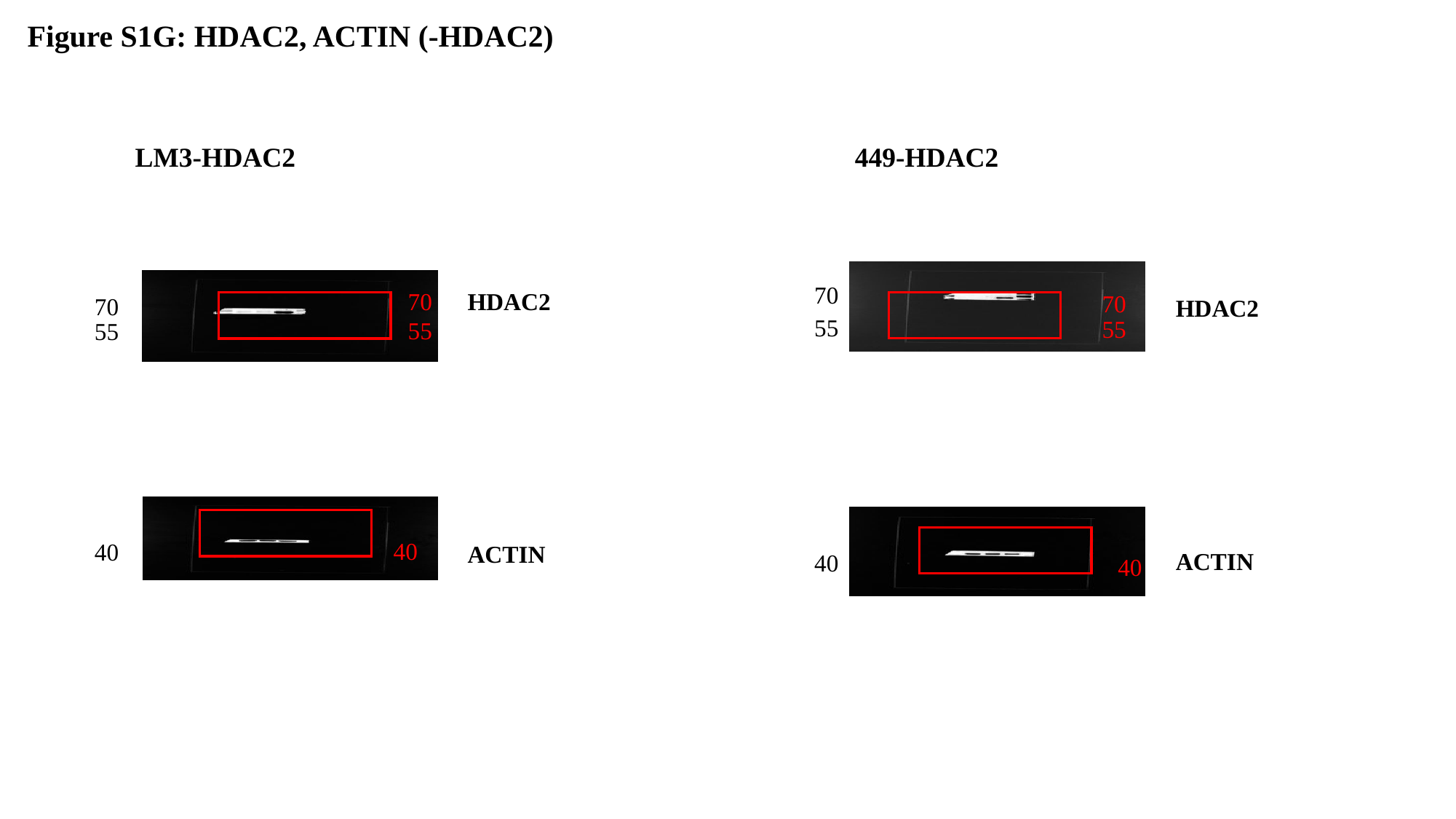

Figure S1G: HDAC2, ACTIN (-HDAC2)
LM3-HDAC2
449-HDAC2
70
HDAC2
70
70
70
HDAC2
55
55
55
55
40
40
ACTIN
ACTIN
40
40

## Slide 17
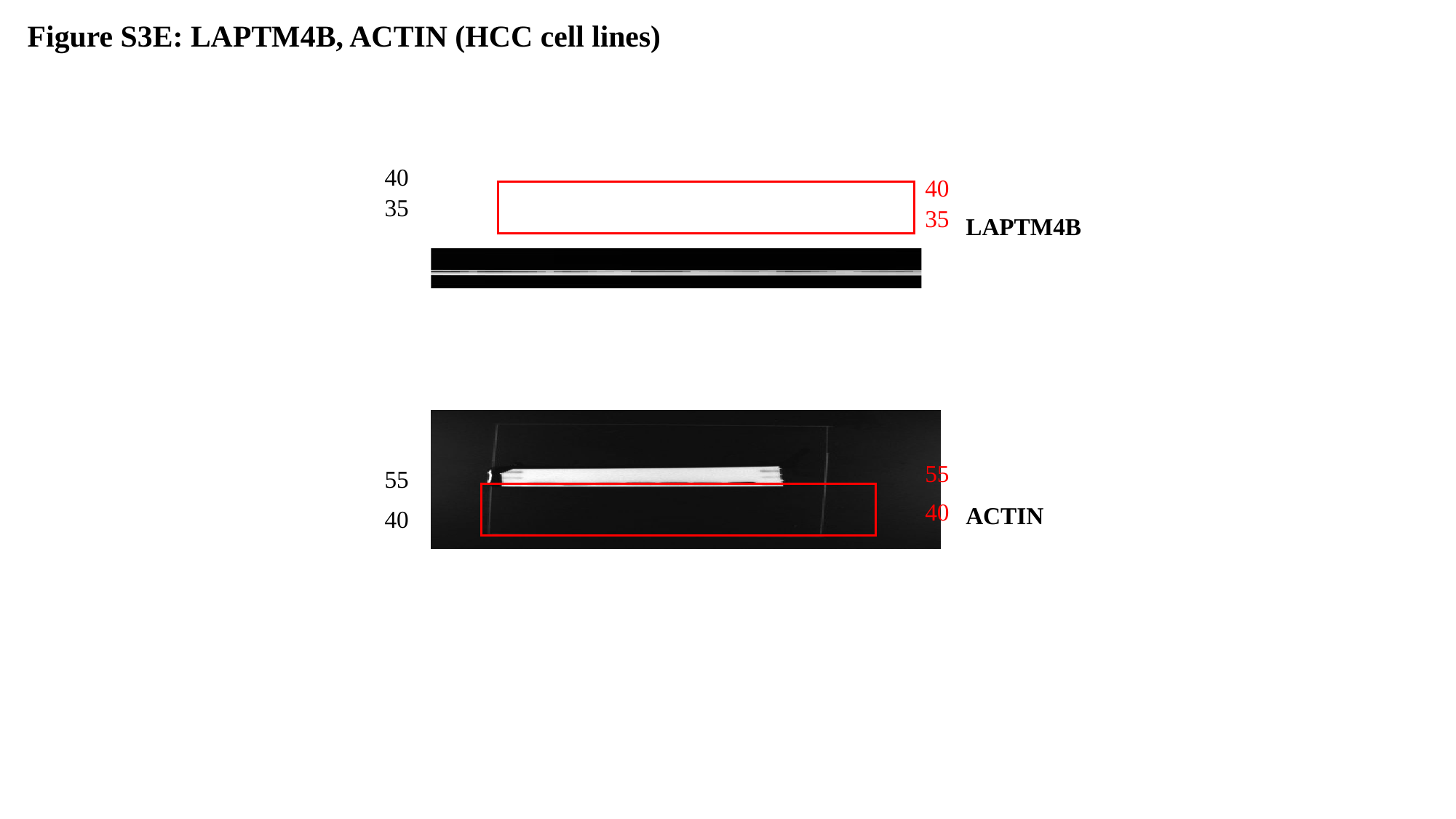

Figure S3E: LAPTM4B, ACTIN (HCC cell lines)
40
40
35
35
LAPTM4B
55
55
40
ACTIN
40

## Slide 18
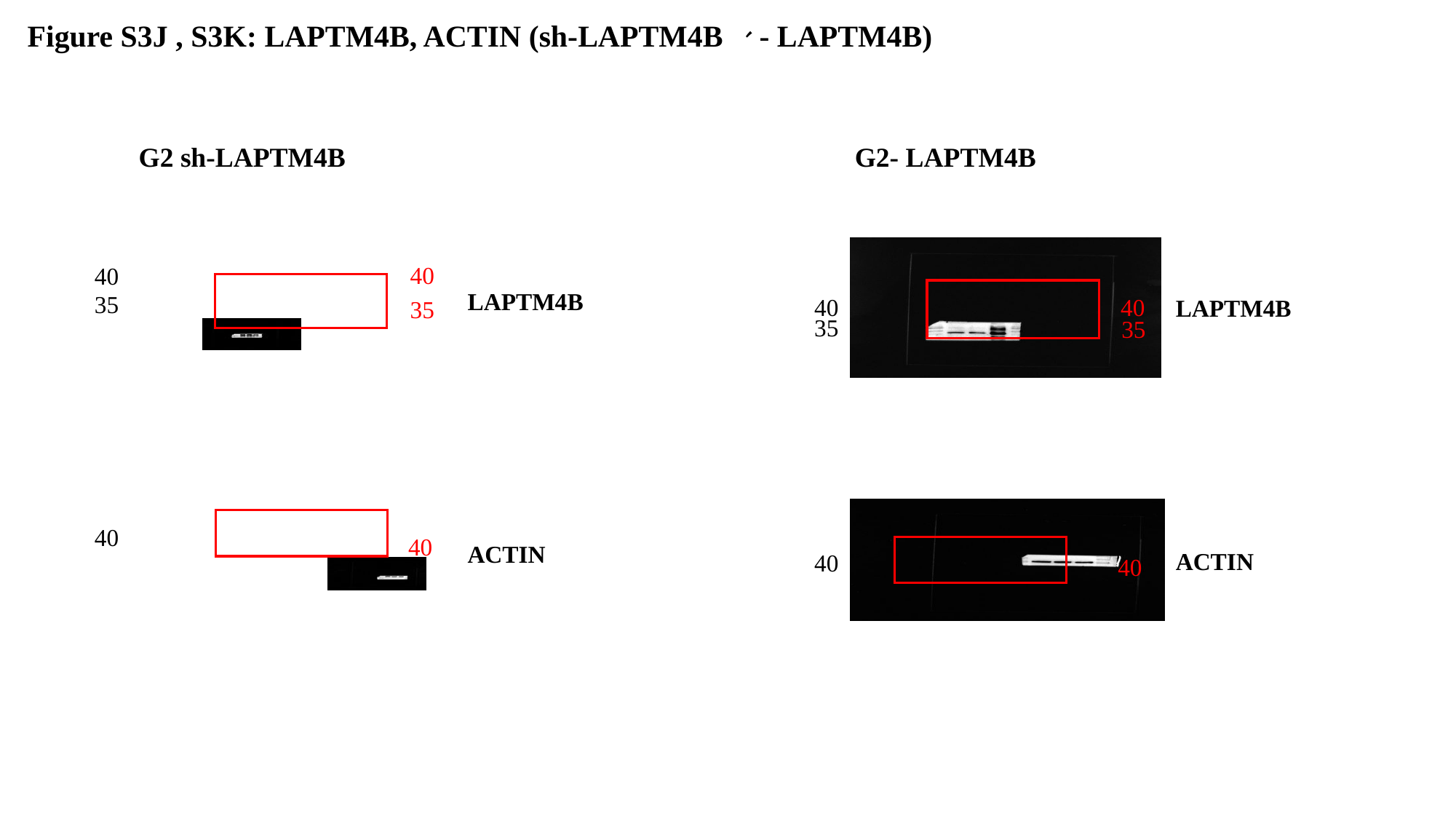

Figure S3J , S3K: LAPTM4B, ACTIN (sh-LAPTM4B 、- LAPTM4B)
G2 sh-LAPTM4B
G2- LAPTM4B
40
40
LAPTM4B
35
40
40
LAPTM4B
35
35
35
40
40
ACTIN
ACTIN
40
40

## Slide 19
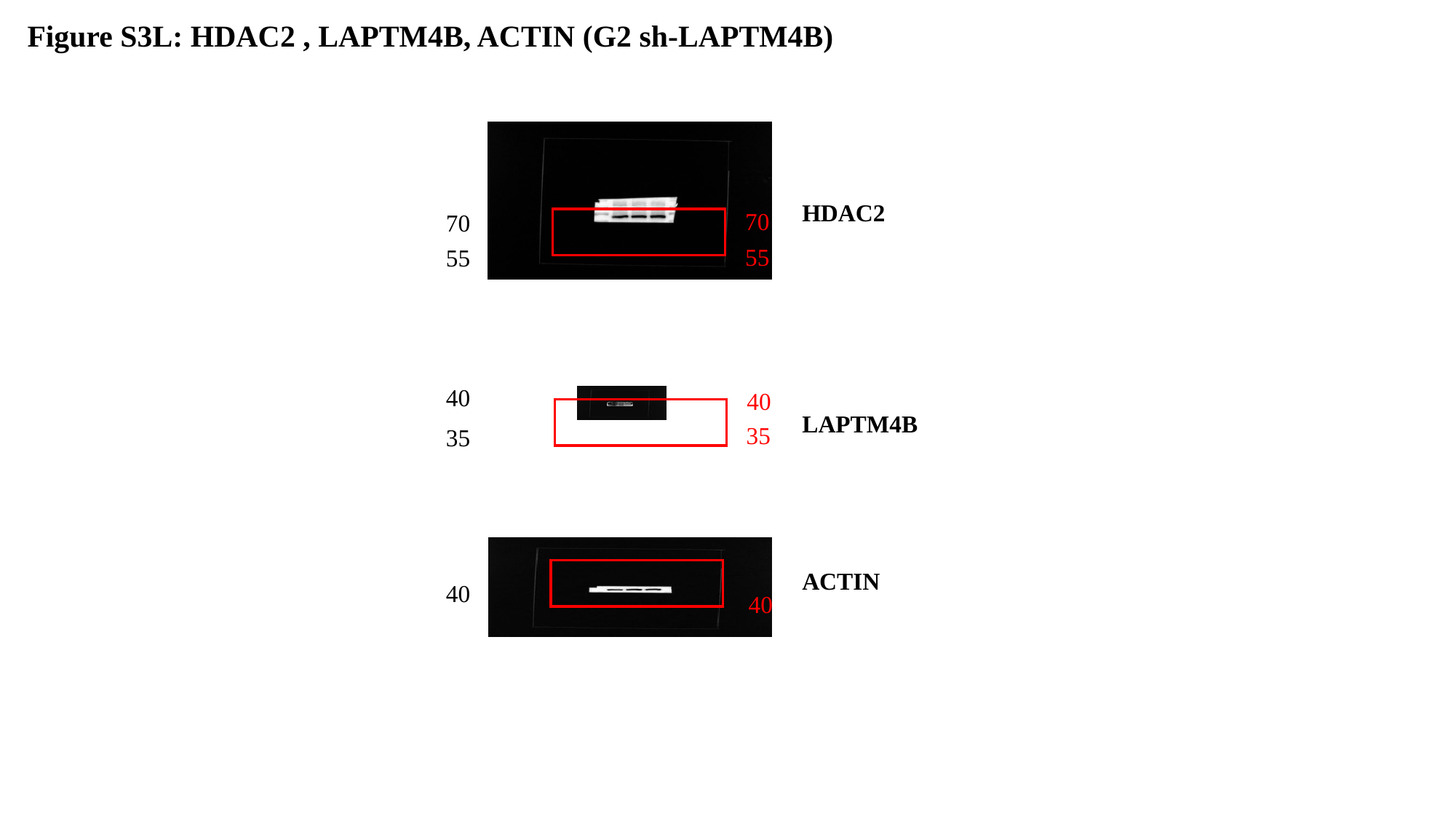

Figure S3L: HDAC2 , LAPTM4B, ACTIN (G2 sh-LAPTM4B)
HDAC2
70
70
55
55
40
40
LAPTM4B
35
35
ACTIN
40
40

## Slide 20
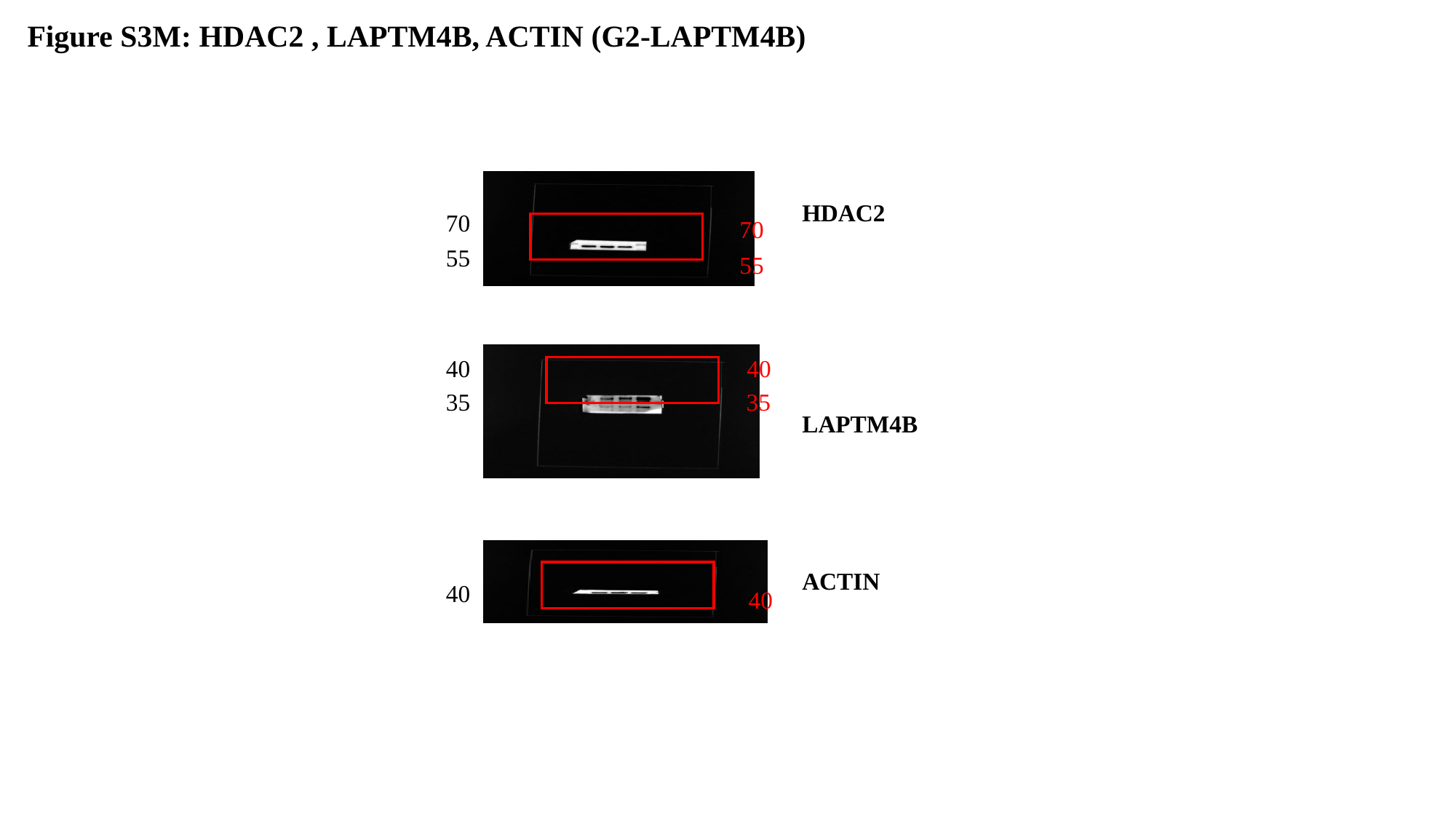

Figure S3M: HDAC2 , LAPTM4B, ACTIN (G2-LAPTM4B)
HDAC2
70
70
55
55
40
40
35
35
LAPTM4B
ACTIN
40
40
